# Diversity of *Colletotrichum* Species Associated with Olive Anthracnose Worldwide

**DOI:** 10.3390/jof7090741

**Published:** 2021-09-09

**Authors:** Juan Moral, Carlos Agustí-Brisach, Maria Carmen Raya, José Jurado-Bello, Ana López-Moral, Luis F. Roca, Mayssa Chattaoui, Ali Rhouma, Franco Nigro, Vera Sergeeva, Antonio Trapero

**Affiliations:** 1 Departamento de Agronomía (DAUCO María de Maeztu Unit of Excellence 2021–2023), Campus de Rabanales, Universidad de Córdoba, Edif. C4, 14071 Córdoba, Spain; cagusti@uco.es (C.A.-B.); ag2raorm@uco.es (M.C.R.); jose.jbello@gmail.com (J.J.-B.); b92lomoa@uco.es (A.L.-M.); ag3rocal@uco.es (L.F.R.); 2 Laboratory of Improvement and Protection of Olive Genetic Resources, Olive Tree Institute, BP 208 Cité Mahrajene, Tunis 1082, Tunisia; mayssa.chattaoui@yahoo.fr (M.C.); ali.rouma@gmail.com (A.R.); 3 Dipartimento di Scienze del Suolo, della Pianta e degli Alimenti, University of Bari Aldo Moro, 70126 Bari, Italy; franco.nigro@uniba.it; 4 School of Science and Health, Western Sydney University, Penrith 2747, Australia; sergeeva@tpg.com.au

**Keywords:** anthracnose, *Colletotrichum* spp., diversity, *Olea europaea*, pathogenicity, phenotype, phylogenetic analysis

## Abstract

Olive anthracnose caused by *Colletotrichum* species causes dramatic losses of fruit yield and oil quality worldwide. A total of 185 *Colletotrichum* isolates obtained from olives and other hosts showing anthracnose symptoms in Spain and other olive-growing countries over the world were characterized. Colony and conidial morphology, benomyl-sensitive, and casein-hydrolysis activity were recorded. Multilocus alignments of ITS, TUB2, ACT, CHS-1, HIS3, and/or GAPDH were conducted for their molecular identification. The pathogenicity of the most representative *Colletotrichum* species was tested to olive fruits and to other hosts, such as almonds, apples, oleander, sweet oranges, and strawberries. In general, the phenotypic characters recorded were not useful to identify all species, although they allowed the separation of some species or species complexes. ITS and TUB2 were enough to infer *Colletotrichum* species within *C. acutatum* and *C. boninense* complexes, whereas ITS, TUB2, ACT, CHS-1, HIS-3, and GADPH regions were necessary to discriminate within the *C. gloesporioides* complex. Twelve *Colletotrichum* species belonging to *C. acutatum*, *C. boninense*, and *C*. *gloeosporioides* complexes were identified, with *C. godetiae* being dominant in Spain, Italy, Greece, and Tunisia, *C. nymphaeae* in Portugal, and *C. fioriniae* in California. The highest diversity with eight *Colletotrichum* spp. was found in Australia. Significant differences in virulence to olives were observed between isolates depending on the *Colletotrichum* species and host origin. When other hosts were inoculated, most of the *Colletotrichum* isolates tested were pathogenic in all the hosts evaluated, except for *C. siamense* to apple and sweet orange fruits, and *C. godetiae* to oleander leaves.

## 1. Introduction

The olive (*Olea europaea* L. subsp. *europaea*) is the most important tree crop worldwide, covering over 11 million hectares, more than the whole of stone fruit species [1]. Most olives are grown near the Mediterranean Sea, especially Spain, Italy, Greece, Tunisia, and Portugal. The excellent adaptation of the olive plant to different conditions has prompted a spread of olive farming to countries where it is not a traditional crop, such as Australia, Brazil, or China [2,3]. Due to this expansion through different areas, the olive plant has been gradually exposed to new pathogens. This situation is particularly striking in olive anthracnose, the most important disease of the fruit.

**Figure 1:**
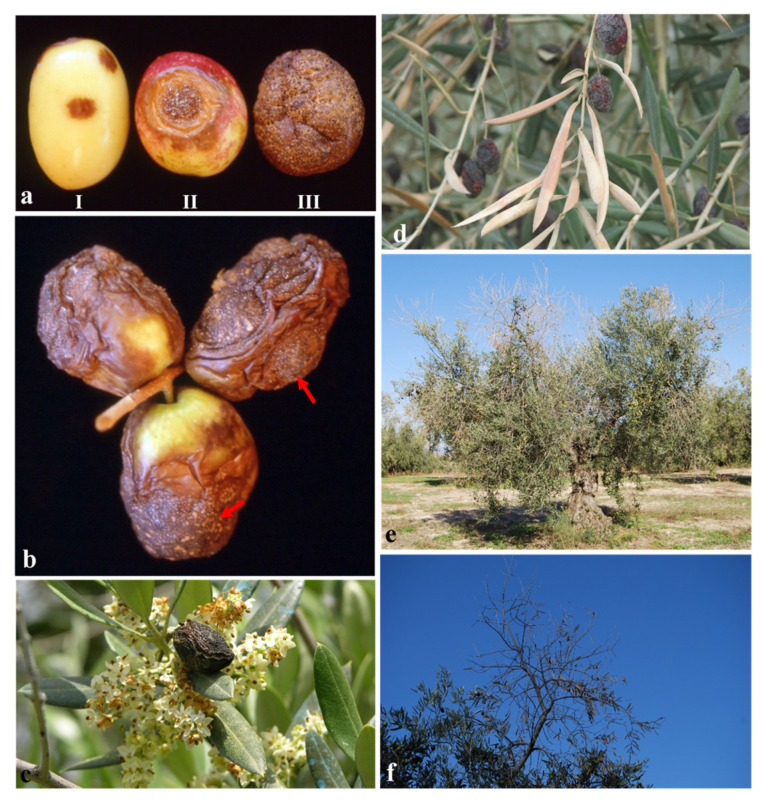
Typical symptoms of olive anthracnose. (**a**) Disease progression on naturally infected olive fruit (I: incipient symptoms; II: depressed, round, and ochre-brown lesions; III: rotted fruit with orange conidial masses produced by *Colletotrichum* spp.); (**b**) detail of orange conidial masses (red arrows) on infected olive fruit; (**c**,**d**) mummified fruit remaining in the tree canopy causing flower and leaf blight; (**e**,**f**) dieback of shoots and branches caused by *Colletotrichum* spp. in olive trees.

Olive anthracnose caused by numerous *Colletotrichum* species causes dramatic losses of fruit yield and oil quality during epidemic years [4,5,6,7]. The pathogen infects through the seasons, but disease symptoms appear at the beginning of ripening when the color of the fruit changes from green to black [6,8]. Typical symptoms are depressed, round, and ochre or brown lesions leading to fruit rot with great orange conidial masses (Figure 1a,b), the “soapy olive” syndrome that gives its name to this disease in Spanish [9]. Subsequently, fruit are mummified (Figure 1c,d) when the temperature falls, relative humidity increases in late autumn-winter, and most of them fall to the soil [6,10]. The pathogen also causes the dieback of olive branches via phytotoxins (Aspergillomarasmine A) produced by the fungus in the rotten fruit (Figure 1e,f) [8,11,12]. Likewise, the pathogen can cause the blight of olive inflorescences, mainly when mummies remain attached to the tree canopy during the flowering [8,12,13]. In addition, the pathogen may act as a secondary invader of injured tissue and can also survive as endophyte or saprophyte. The ability to survive and multiply in the absence of symptoms may explain why anthracnose fungi often cause unexpected crop losses in olives [12,14].

The causal agent of olive anthracnose was described for the first time in Portugal by de Almeida [15] as *Gloeosporium olivarum*. Subsequently, this species was reclassified as *Colletotrichum gloeosporioides* (anamorph of *Glomerella cingulata*) after reviewing the *Gloeosporium* genus by von Arx [16]. Later, the species *C. gloeosporioides* was considered a heterogeneous species complex affecting about 300 plant species [17]. Currently, over 1000 epithets are listed in Mycobank [18] under *Colletotrichum*, which comprises 248 accepted species, most of them grouped into 14 species complexes [19].

As with many other crops, olives can be affected by a wide range of *Colletotrichum* species [20,21,22]. To date, a total of 14 *Colletotrichum* spp. have been associated with olive anthracnose over the world. These species belong to three *Colletotrichum* complexes: *C. acutatum*, *C. boninense*, and *C*. *gloeosporioides* [5,13,23,24]. Among them, the species *C. acutatum sensu stricto* (from now on *C. acutatum*), *C. fioriniae*, *C. godetiae*, *C. nymphaeae*, *C. rhombiforme*, and *C. simmondsii*, all of them belonging to the *C. acutatum* species complex, are currently considered the major pathogens of this genus [5,13,24]. While several *Colletotrichum* species can be found in an olive-growing area, there is usually one dominant one and some secondary [7]. For example, the species *C. nymphaeae* is dominant in the olive orchards of Portugal [4,25], while *C. godetiae* is the prevalent species in several Mediterranean countries such as Greece, Montenegro, and Spain [6,7,24,26].

In southern Italy, Faedda et al. [26] found that the *Colletotrichum* population of olive trees consisted of mainly those dominated by *C. clavatum*. However, Damm et al. [27] considered *C. clavatum* as a synonym of the older species *C. godetiae*. Later, different studies revealed a wide distribution of *C. acutatum* and *C. godetiae* together with four species—*C. aenigma*, *C. gloeosporioides*, *C. cigarro* (*C. gloeosporioides* especies complex), and *C. karstii* (*C. boninense* complex)—associated with olive anthracnose in southern Italy [23,24,28,29]. Consequently, Mosca et al. [28] and Schena et al. [24] have hypothesized that *C. acutatum* is an emerging olive pathogen in Italy. This latter species has also been reported to cause olive anthracnose in Australia, Brazil, Greece, Portugal, South Africa, Tunisia, and Uruguay [4,5,13,30,31,32].

Furthermore, other species belonging to the *C. gloeosporioides* species complex have been described in Australia (*C. siamense* and *C. theobromicola*), Montenegro (*C. queenslandicum*), and Uruguay (*C. alienum* and *C. theobromicola*) as associated with olive fruit [13,23]. Nevertheless, the pathogenicity of several of these species (*C. aenigma*, *C. cigarro*, *C. karstii*, *C. queenslandicum*, and *C. siamense*) on olive fruit is still uncertain, suggesting that their role in the fruit infection could be secondary [5].

From the first descriptions of *C. acutatum sensu lato* and *C. gloeosporioides s. l*. as the causal agents of the olive anthracnose in 1999 [33], many studies focused on aetiology have been conducted mainly in Italy and Portugal, generating relevant knowledge about the diversity of *Colletotrichum* species associated with the disease [4,23,24,28,34]. In the case of Spain, the etiological knowledge about olive anthracnose is much more limited and suggests that there are two prevalent species, with *C. godetiae* being dominant [7]. Therefore, more etiological studies are necessary to elucidate the diversity of *Colletotrichum* species involved in the olive anthracnose. Furthermore, differences in virulence among *Colletotrichum* species, and isolates of the same species, have also been described in different woody crops [5,35,36,37,38]. However, the number of species tested is still slight, and broader studies on the pathogenicity of *Colletotrichum* spp. causing olive anthracnose are necessary.

During these last two decades, many *Colletotrichum* isolates from olive fruit showing anthracnose symptoms in orchards located in the Iberian Peninsula, Spain, and Portugal were studied in our laboratory. Both Spain and Portugal produce around 65% of the global supply of olive oil [1]. In parallel, many *Colletotrichum* isolates from olives or other susceptible hosts to anthracnose from Australia, Brazil, California, Greece, Portugal, Italy, Tunisia, and Uruguay have been studied in close collaboration with different international research groups. Thus, the main goal of the present study was to characterize a vast collection of *Colletotrichum* isolates obtained from olives and other hosts showing anthracnose symptoms in Spain and other olive-growing countries. To this end, in the present study, we combined different techniques for characterization of the *Colletotrichum* population affecting olive fruit around the world, including morphological characteristics [4,5,16,26,31], physiological traits including tolerance to fungicides and enzymatic activity [4,5,7,33], and molecular tools [4,19,23,27]. Therefore, the specific objectives of this study were (*i*) to obtain a wide collection of *Colletotrichum* isolates representative of the geographic origin described above and from different hosts showing anthracnose symptoms; (*ii*) to characterize them based on phenotypic (colony and conidial morphology; and benomyl-sensitive and casein-hydrolysis tests) and molecular characters (multilocus alignments and phylogenetic analyses); (*iii*) to determine the pathogenicity of *Colletotrichum* isolates to olive fruit; and (*iv*) to evaluate cross-pathogenicity on different *Colletotrichum* hosts. Elucidating the biodiversity of *Colletotrichum* species causing olive anthracnose is essential for a better understanding of the aetiology and epidemiology of the most critical fruit disease of this legendary crop.

## 2. Materials and Methods

### 2.1. Collection of Fungal Isolates

Olive fruit samples showing symptoms of anthracnose were collected from many commercial orchards from 1998 to 2016. Symptomatic fruit were collected from different provinces across Spain, emphasizing the orchards located in the Andalusia region of southern Spain, the world’s leading olive-producing region. Many other samples were collected from commercial orchards situated in southern Portugal, where olive anthracnose is endemic [34,39]. Isolations were made from affected fruit with the typical anthracnose lesions. Diseased fruit were surface disinfested with commercial bleach (Cl at 50 g L^–1^) at 10% (*v*/*v*) in sterile water for 1 min, and air-dried on sterile filter paper for 30 min. Affected tissues were cut with a sterile scalpel and plated on potato dextrose agar (PDA) (Difco Laboratories^®^, Detroit, MI, USA) acidified with lactic acid (2.5 mL of 25% [*v*/*v*] per liter of medium) to minimize bacterial growth (APDA). When the affected fruit tissues showed abundant pathogen sporulation, masses of conidia were removed using a sterile needle and cultured in Petri dishes on APDA. Petri dishes were incubated at 23 ± 2 °C under a 12-h daily photoperiod of cool fluorescent light (350 μmol m^–2^ s^–1^) for 5 days. Single-spore isolates were prepared before use in further experiments using serial dilutions [40]. Moreover, *Colletotrichum* isolates recovered from olive fruit showing anthracnose symptoms in Australia, Brazil, California (the USA), Greece, Italy, Tunisia, and Uruguay were also included in this study as collaboration with several international research groups from those major olive-growing regions of the world (Table 1). All the isolates were maintained on colonized PDA into sterile plastic tubes with sterile paraffin oil (Panreac Química SA, Barcelona, Spain) at 4 °C in darkness for long-term storage in the fungal collection of the Department of Agronomy at the University of Cordoba (UCO; Spain).

### 2.2. Phenotypic Characterization

#### 2.2.1. Colonies and Conidial Morphology

Thirty-eight representative isolates belonging to *C. acutatum* (27 isolates), *C. boninense* (two isolates), and *C. gloeosporioides* (nine isolates) species complexes (Table 1) were used to study mycelium colony and conidium morphology. To this end, all the isolates were grown on PDA (Difco^®^ Laboratories, Detroit, MI, USA) for two weeks at the same incubation conditions described above. There were three replicated Petri dishes per isolate.

Characteristics of mycelia (texture, density, color, and zonation) were recorded by visual observations on 7-day-old colonies [41]. For all isolates, color was determined using a color scale [42]. For conidial measures, conidial masses removed from the margin of 10-day-old colonies were placed on slides with a drop of 0.005% acid fuchsine in lactoglycerol (1:1:1 lactic acid, glycerol, and water) and covered with a coverslip. For each isolate and replicate Petri dish, the size, and the shape of 50 conidia were measured utilizing a Nikon Eclipse 80i microscope (Nikon Corp., Tokyo, Japan) at 400× magnification. The conidial size was determined by measuring its length and width, and the length/width ratio was calculated. According to their shape, conidia were classified into three categories: (0) Conidia with two rounded ends (ellipsoid); (1) Conidia with one rounded end and the other acute (clavate); and (2), Conidia with two acute (sharp) ends (fusiform). Data were expressed as a percentage (%) of each type of conidium.

#### 2.2.2. Benomyl-Sensitive Assay

Twenty-seven representative isolates belonging to *C. acutatum* (16 isolates), *C. boninense* (two isolates), and *C. gloeosporioides* (nine isolates) species complexes were used to evaluate their sensitivity to benomyl by in vitro sensitivity assay (Table 1). Based on previous studies [4,43], and our preliminary trials using isolates of *C. acutatum* and *C. gloeosporioides* species complexes, we determined a threshold of 5 µg of benomyl per milliliter to differentiate sensitive and tolerant isolates to this fungicide. Thus, mycelial plugs (5 mm in diameter) obtained from the margins of 7-day old actively growing colonies on PDA were transferred to Petri dishes with PDA amended with 5 µg mL^−1^ of benomyl (benomyl 50%, WP, Adama Agriculture, Madrid, Spain). Mycelial plugs of each isolate were plated on non-amended PDA as control. All Petri dishes were incubated under the described conditions. There were three replicated Petri dishes per isolate and treatment (benomyl and control), and the experiment was conducted twice.

The evaluation was performed at 7 days, measuring the largest and smallest diameters of each colony. For each isolate, the inhibition percentage (%) was calculated by comparing the growth on PDA and on PDA amended with benomyl. Data from repetitions of the experiment were combined after checking for homogeneity of variances of the experimental error of the two replicated experiments by the *F* test. Subsequently, analysis of variance (ANOVA) was conducted using a randomized complete block design with the two repetitions of the experiment as blocks, fungal isolate as the independent variable, and inhibition percentage as the dependent variable. Mean comparisons were made using Tukey’s honestly significant difference (HSD) test [44]. Data were analyzed using Statistix 10 [45].

#### 2.2.3. Casein-Hydrolysis Assay

The 27 *Colletotrichum* isolates, previously studied according to their sensitive/tolerance benomyl fungicide, were also characterized according to their ability to hydrolyse the casein. Thus, the 27 *Colletotrichum* isolates were transferred, as described previously, to hydrolyse casein medium (CHM). We formulated the CHM media using a 15% milk powder solution (Sveltesse Nestle^®^, Esplugues de Llobregat, Barcelona, Sapin) in deionized water sterilized at 120 °C for 15 min, and 20 mL of the sterile milk solution was added to 980 mL of sterilized Water Agar (WA; Biokar-Diagnostics, Allonne, France) before solidification (around 50 °C) and homogenized for 2 min using a magnetic rotor (Agimatic-N, JP-Selecta, Barcelona, Spain).

A 5-mm diameter plug of each *Colletotrichum* isolate was plated to Petri dishes with CHM and incubation for 5 days as described for the benomyl sensitivity assay. For each isolate, mycelial plugs were plated on non-amended PDA as control. We visually determined the presence or absence of the hydrolysis halo surrounding the *Colletotrichum* colony growing on CHM media. There were three replicated Petri dishes per isolate and treatment (milk powder suspension and control), and the experiment was conducted twice.

### 2.3. Molecular Characterization

#### 2.3.1. DNA Extraction, PCR, Sequencing, and Nucleotide Alignment

Genomic DNA was extracted from 100 mg of mycelium of the 185 *Colletotrichum* isolates growing on PDA (Table 1). Mycelial tissues were ground using a FastPrep^®^-24 grinder machine (MP Biomedicals, Irvine, CA, USA). Subsequently, DNA extractions were carried out using E.Z.N.A.^®^ Fungal DNA Mini Kit (OMEGA bio-tek, Norcross, GA, USA) following the manufacturer’s instructions. The concentration and purity of extracted DNA were determined by means of MaestroNano^®^ spectrophotometer (MaestroGen, Hsinchu City, Taiwan).

Six genomic areas, 5.8S nuclear ribosomal gene with two flanking internal transcribed spacers (ITS), beta-tubulin (TUB2), actin (ACT), partial sequences of the chitin synthase 1 (CHS-1), histone 3 (HIS3), and a 200-bp intron of the glyceraldehyde-3-phosphate dehydrogenase (GAPDH), were amplified and sequenced. For that, the following primer pairs were correspondingly used: ITS4 and ITS5 [46], Bt-2a and Bt-2b [47], ACT-512F and ACT-783R [48], CHS-354R and CHS-79F [48], CYLH3F and CYLH3R [49], and GDF1 and GDR1 [50]. Additionally, to infer the identity of fungal isolates belonging to the *C. gloeosporioides* complex, the intergenic region between Apn2 and Mat1-2 genes (ApMat) was also amplified and sequenced with the primer pair AMF1 and AMR1 [51].

PCR amplifications were performed in a MyCycler^TM^ Thermal Cycler (BIO-RAD) in a total volume of 25 µL. All PCRs mixture contained 5 µL of 5×MyTaq reaction buffer, 0.13 µL of MyTaq DNA polymerase (Bioline), and 20 ng of genomic DNA template. Additionally, 0.2 µM of each primer was added for the ACT, CHS-1, HIS3, and GADPH PCRs, and 0.4 µM of each primer for ITS, TUB2, and ApMat PCRs. Negative control was included in all PCRs using ultrapure water instead of DNA. The PCRs cycling programs were conducted as follows: an initial denaturation at 95 °C for 5 min, followed by 40 cycles of 95 °C for 15 s, annealing for 15 s and 72 °C for 10 s, and a final extension at 72 °C for 7 min. The annealing temperatures used were: 48 °C for ITS, 52 °C for GAPDH, CHS-1, HIS3, TUB2, and ACT, and 55 °C for ApMat. All PCRs were stopped at 4 °C.

Amplification products were checked by electrophoresis in 1.7% (wt/vol) agarose gel stained with RedSafe (Intron Biotechnology, Sagimakgol-ro Joongwon-gu Seongnam-Si Korea, Republic of South) and visualized under ultraviolet light. DNA gTP-Ladder (gTPbio) was used for electrophoresis as DNA size markers. Single-band products were purified using MEGAquick-spin^TM^ Total Fragment DNA Purification kit (INTRON Biotechnology), following the manufacturer’s instructions. Subsequently, purified PCR products were sequenced in both forward and reverse directions by the Central Service Support Research (SCAI) at the University of Córdoba (Spain).

Generated sequences were assembled and edited using the software SeqMan^®^ v. 7.0.0. (DNASTART LaserGen, Madison, WI, USA). Consensus sequences for all isolates were compiled into a single file (Fasta format) and were deposited in GenBank (http://www.ncbi.nlm.nih.gov/genbank/ accessed on 1 Augest 2021) (Table 1).

#### 2.3.2. Phylogenetic Analyses and Species Delimitation

All consensus sequences were blasted against the NCBIs GenBank nucleotide database to determine the closest relative species of *Colletotrichum* to our isolates. In total, 185 isolates of *Colletotrichum* were included in the molecular phylogenetic analyses. Additionally, sequences from 70 species of *Colletotrichum* (90 isolates in total) were downloaded from GenBank and included in the analysis as reference sequences or outgroups (Table 1). Reference sequences were selected based on their high similarity with our query sequences using MegaBLAST and were added to the data set and aligned using CLUSTAL W v. 2.0.11 [52].

A Neighbour-Joining (NJ) analysis was performed individually for each genomic area using the Maximum Composite Likelihood method and 2000 bootstrap replications to determine whether the sequence datasets were congruent and combinable. Tree topologies of 70% reciprocal bootstrap generated individually for each locus were compared visually. Because no supported nodes were in conflict, the data of different loci were combined into single concatenated datasets. Three different datasets were analyzed to compare and identify our *Colletotrichum* isolates correctly. For a first identity approach, one phylogeny was constructed using a combination of ITS and TUB2 sequences (*dataset I*). This phylogeny consisted of 40 taxa of the *Colletotrichum* genus including species belonging to *C. acutatum*, *C. boninense,* and *C. gloeosporioides* species complexes, among other *Colletotrichum* spp., with *C. dracaenophilum* (CBS 118199) as an outgroup. Subsequently, a second phylogeny was performed by multilocus alignment of ITS, TUB2, ACT, CHS-1, HIS-3, and GADPH sequences (*dataset II*) to identify our isolates [27]. This second phylogeny consisted of 41 taxa of the *Colletotrichum* genus including species belonging to *C. acutatum*, *C. boninense,* and *C. gloeosporioides* species complexes, with *C. dracaenophilum* (CBS 118199) again as an outgroup. A third multilocus alignment combining the ITS, TUB2, and ApMat sequences (*dataset III*) was performed for inferring organismal phylogeny of 14 isolates belonging to the *C. gloeosporioides* species complex [53,54].

For multilocus alignments, phylogenetic analyses were conducted by Bayesian Inference (BI) and Maximum Parsimony (MP). The MP trees were obtained using the Tree-Bisection-Regrafting (TBR) algorithm with search level 1, in which the initial trees were obtained by the random addition of sequences (10 replicates). All positions containing gaps and missing data were eliminated. A set of 2000 bootstrap replications evaluated the robustness of the generated trees. Tree length (TL), consistency index (CI), retention index (RI), rescaled consistency index (RC), and homoplasy index (HI) were recorded. BI analyses were performed with MrBayes v.3.2.6 [55], which uses Markov Chain Monte Carlo to approximate the posterior probability of trees. Two analyses with four chains each were run at the same time, for 1 × 10^7^ generations, sampled every 100 generations, and starting from a random tree topology. The “temperature” parameter was set to 0.2. For the consensus tree, the first 25% of the saved trees were discarded as the burn-in phase of the analysis. Each of the individual genes and a combined data set were aligned, adjusted manually, and analyzed by NJ or MP using MEGA v.7 [56]. In BI and NJ analyses, the best evolutionary model for each gene partition was also determined by MEGA v.7. The genes were concatenated in a single nucleotide alignment using Phylogenetic Data Editor (PhyDE-1).

### 2.4. Pathogenicity Test

#### 2.4.1. Pathogenicity on Olive Fruit

The following *Colletotrichum* isolates were evaluated according to their pathogenicity on olive fruit: *C. acutatum* isolates from olive fruit (Col-193 and Col-256) and almond fruit (Col-536); *C. fioriniae* isolate from olive fruit (Col-172); *C. gloeosporioides* from olive fruit (Col-41) and sweet orange fruit (Col-69); *C. godetiae* from olive fruit (Col-30, Col-57, Col-88, Col-508, Col-515, and Col-519) and almond fruit (Col-522); *C. karstii* from sweet orange fruit (Col-79); and *C. nymphaeae* isolates from olive fruit (Col-42 and Col-506) and strawberry fruit (Col-84 and Col-86) (Table 1). Violet (color class 3) olive fruit of the highly susceptible cv. Hojiblanca were collected from olives growing in the World Olive Germplasm Bank (WOGB), belonging to the IFAPA located in the Córdoba province [57]. Before inoculation, the olive fruits were washed and surface-disinfested according to Moral et al. [9]. Surface-disinfected olive fruits were placed in moist chambers (plastic containers, 22 × 16 × 10 cm) at 100% relative humidity (RH) and inoculated by spraying them up run-off with a conidial suspension adjusted with a haemocytometer to 10^5^ conidia mL^−1^. After inoculation, humid chambers were incubated at 23 ± 2 °C with a 12-h photoperiod. Additionally, olive fruit sprayed with sterile distilled water were included as a control. There were three replicated humid chambers per isolate and 20 fruits per humid chamber, and the experiment was conducted twice. A completely randomized design was used with fungal isolates as the independent variable and moist chambers as replications. The pathogens were re-isolated from the olive fruit as described above.

#### 2.4.2. Pathogenicity on Other Hosts

*Colletotrichum godetiae* isolates from olive fruit (Col-9 and Col-57), *C. gloeosporioides* from sweet orange fruit (Col-69), *C. karstii* from sweet orange fruit (Col-79), *C. nymphaeae* from olive fruit (Col-42), and *C. siamense* from strawberry fruit (Col-44) were selected to evaluate their pathogenicity on different hosts (Table 1). Fruits of almond (*Prunus dulcis* (Mill.) D.A.Webb) cv. Guara, apple (*Malus domestica* Borkh.) cv. Golden Delicious, sweet orange (*Citrus sinensis* L.) cv. Lanelate, and strawberry (*Fragaria* × *Ananassa* L.) cv. Camarosa, as well as leaves of oleander (*Nerium oleander* L.) were selected for this assay. Plant material was washed, and surface disinfested as described above. The pathogenicity of the six *Colletotrichum* isolates was evaluated by independent inoculation on the different hosts. Thus, almond, apple, olive, and strawberry fruits were inoculated by surface deposition of one mycelial plug (9 mm in diameter) per fruit pierced with a sterile needle, according to Moral et al. [9]. Oleander leaves were inoculated by the same method, but in this case, three mycelial plugs (7 mm in diameter) were deposited per leaf. Inoculated fruits and leaves were incubated in moisture chambers at 23 ± 2 °C with a 12-h photoperiod. Additionally, non-inoculated fruits or leaves treated with PDA plugs were included as a control. There were three replicated humid chambers per isolate-host combination, 10 fruits or leaves per humid chamber, and the experiment was conducted twice. A completely randomized design was used with fungal isolates and host as the independent variable and moist chambers as replications. The pathogens were re-isolated from the fruits and leaves as described above.

#### 2.4.3. Disease Assessment and Data Analysis

Disease severity (DS) in inoculated olive and almond fruits was evaluated weekly until most of the fruit achieved the maximum value (approx. 14 and 21 days for olive and almond fruits, respectively). DS was assessed using a 0–5 rating scale: (0) no symptoms; (1) 1–25% of the fruit surface affected; (2) 26–50%; (3) 51–75%; (4) >75%; and (5) 100% [9]. A disease severity index (DSI) was calculated in each replication using the following formula: *DSI* = [(Σ*n_i_* × *i*)/(*N* × 5)] × 100, where *i* represents a severity (zero to five), *n_i_* is the number of fruits with severity *i*, *N* is the total number of fruits, and five is the highest value of the severity rating scale. For the rest of the hosts, the largest and smallest diameters of lesions were measured weekly, and mean data were converted to the radial growth rate (mm day^−1^). DS of the inoculated fruits of apple, sweet orange, and strawberry, and in leaves of oleander was evaluated weekly until most of the fruits or leaves reached 90–100% of their surface affected (approx. 21, 41, 12, and 18 days for apple, sweet orange, strawberry, and oleander, respectively). In all cases, relative areas under the disease progress curve (RAUDPC) were calculated using the trapezoidal integration of DSI values over time. RAUDPC data from the two runs of the experiment were subjected to analysis of variance (ANOVA). The non-pathogenic isolates were excluded from the statistical analysis. The RAUDPC data were logarithmically transformed when necessary to the homogeneity of variances or normality. When ANOVA showed significant differences for each host, means were compared according to Tukey’s honestly significant difference (HSD) test at *p =* 0.05 [44]. Data were analyzed using Statistix 10 [45].

## 3. Results

### 3.1. Collection of Fungal Isolates

In total, 137 *Colletotrichum* isolates were obtained from different hosts across the Iberian Peninsula: 83 of them isolated from olive trees in Portugal, and 54 of them isolated from olives and other hosts in Spain. Forty-six of the Spanish isolates were obtained from olives located in the four major olive-producing regions (Andalusia, Extremadura, Catalonia, and Valencia; located at Southern, South-western, North-eastern, and Eastern Spain, respectively). The other eight Spanish isolates were recovered from almond (two isolates), *Citrus* (two isolates), *Pistacia terebinthus* (one isolate), and strawberry (three isolates). In addition to Iberian isolates, we included 16 isolates from Australia, 1 isolate from Brazil, 5 isolates from California, 6 isolates from Greece, 12 isolates from Italy, 5 isolates from Tunisia, and 3 isolates from Uruguay obtained from affected olive fruit (Table 1).

### 3.2. Phenotypic Characterization

#### 3.2.1. Colonies and Conidial Morphology

**Figure 2:**
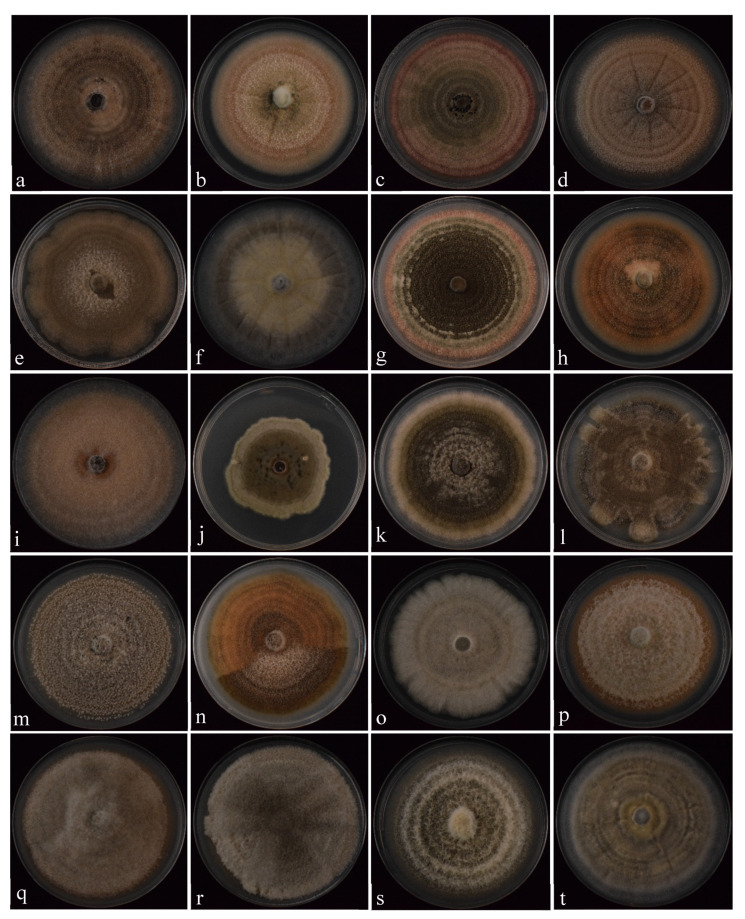
Variability in the colonies of representative *Colletotrichum* isolates belonging to the following species complexes: (**a**–**l**) *Colletotrichum acutatum*; (**m**,**n**) *C. boninense* and (**o**–**t**) *C. gloeosporioides*. Colonies were grown on PDA for 14 days at 25 ± 2 °C under a 12-h daily photoperiod of cool fluorescent light (350 μmol m^–2^ s^–1^). (**a**–**d**) *C. acutatum* ((**a**) Col-166, (**b**) Col-175, (**c**) Col-208, (**d**) Col-536); (**e**–**h**) *C. fioriniae* ((**e**) Col-172, (**f**) Col-693, (**g**) Col-695, (**h**) Col-696); (**i**,**j**) *C. godetiae* ((**i**) Col-88, (**j**) Col-522); (**k**) *C. nymphaeae* Col-42); (**l**) *C. simmondsii* Col-169 (**m**) *C. boninense* Col-178; (**n**) *C. karstii* Col-79; (**o**,**p**) *C. alienum* ((**o**) Col-211, (**p**) Col-214); (**q**) *C. gloeosporioides* Col-69; (**r**,**s**) *C. siamense* ((**r**) Col-44, (**s**) Col-160); (**t**) *C. theobromicola* Col-200.

Most *Colletotrichum* colonies were similar regarding texture and density characteristics with abundant aerial mycelium with regular margins. However, the Australian isolates Col-166, Col-200, Col-152, and Col-214 had colonies with lobulated margins. The growth pattern of all colonies was radial with concentric circles. Nevertheless, the colonies showed a broad variation in color, mainly white, whitish to dark gray, and pinkish-orange being the most common colors observed. Thus, colony color was helpful to discriminate color sub-groups. In general, colonies of all *C. godetiae* isolates were gray (from dark to light gray), colonies of *C. acutatum* isolates showed pinkish-orange tones, and *C. fioriniae* and *C. gloeosporioides* isolates were light gray. In particular, the isolate *C. siamense* Col-44 from strawberry showed a distinctive greenish-gray colony color. However, the rest of the *Colletotrichum* spp. isolates showed colonies with many variations in color within the same species, so it was impossible to establish a relationship between species and the color of their colonies (Table 2; Figure 2).

The average length of the conidia ranged between 8.3 and 14.8 μm for *C. acutatum* isolate Col-175 and *C. gloeosporioides* Col-41, respectively. The average width varied from 2.7 to 5.1 μm for *C. acutatum* isolate Col-175 and for the isolates *C. godetiae* Col-30 and *C. gloeosporioides* Col-69, respectively. In general, the conidia were hyaline, varying in type (ellipsoid, clavate, and fusiform) between isolates within species complex or even within the same fungal species. Isolates belonging to *C. acutatum* species complex had the three types of conidia. Isolates of *C. fioriniae* and *C. nymphaeae* showed fusiform and clavate conidia, respectively. Most isolates identified as *C. godetiae* showed clavate conidia, except isolates Col-88 and Col-522, which showed ellipsoid and fusiform conidia, respectively. *Colletotrichum simmondsii* isolate Col-169 showed fusiform conidia. Concerning the isolates belonging to the *C. boninense* complex, differences in the type of conidia were also observed between species. For example, *C. boninense* isolate Col-178 showed clavate conidia, while *C. karstii* isolate Col-79 showed ellipsoid conidia. Finally, most isolates belonging to the *C. gloeosporioides* complex showed ellipsoid conidia, except two isolates identified as *C. siamense* (isolates Col-44 and Col-184), which showed clavate conidia (Table 2).

#### 3.2.2. Benomyl Sensitive Assay

Wide variability in mycelial growth rate was observed among the *Colletotrichum* isolates grown on PDA amended with 5 µg mL^−1^ of benomyl. In general, *Colletotrichum* isolates developed lower aerial mycelium and greater conidial production than those in the presence of the fungicide. There were significant differences (*p* < 0.001) for mycelial growth inhibition between isolates. According to their sensitivity to benomyl, the *Colletotrichum* isolates could be grouped into two groups (moderately and highly sensitive). The moderately sensitive group only included isolates belonging to *C. acutatum* species complex, whose percentages of inhibition ranged from 33.5% to 71.1% for *C. acutatum* isolate Col-166 and *C. fioriniae* isolate Col-172, respectively, both from olive trees in Australia (Table 2). The highly sensitive group was formed by isolates belonging to *C. boninense* and *C. gloeosporioides* species complexes, whose percentages of mycelial growth inhibition ranged from 93.8% to 100% for *C. siamense* isolate Col-160 (from olive fruit, Australia) and *C. gloeosporioides* isolate Col-41 (from olive fruit, Spain), respectively. However, no benomyl-resistant *Colletotrichum* isolates were observed in any case.

#### 3.2.3. Hydrolysis-Casein Assay

Seventeen out of the 26 tested *Colletotrichum* isolates caused a casein hydrolysis halo surrounding their colonies in CHM that was observable at 1 day of incubation. At 5 days of incubation, *Colletotrichum* isolates were classified as able (+ or ++ for the width of halo ≤ 2 or > 2 mm, respectively) or not able (-) to hydrolyze casein. This phenotypic characteristic was also helpful to discriminate isolates between *Colletotrichum* species complexes, but with some exceptions. Thus, all the isolates belonging to the *C. acutatum* species complex could hydrolyze casein, except *C. fioriniae* isolate Col-172 from olive trees in Australia. Most isolates belonging to *C. boninense* and *C. gloeosporioides* species complexes could not hydrolyze casein, except three isolates within the *C. gloeosporioides* species complex (*C. alienum* isolate Col-211, *C. siamense* isolates Col-184, and Col-187, all of them from olive trees in Australia) (Table 2).

### 3.3. Molecular Characterization. Phylogenetic Analyses

**Figure 3:**
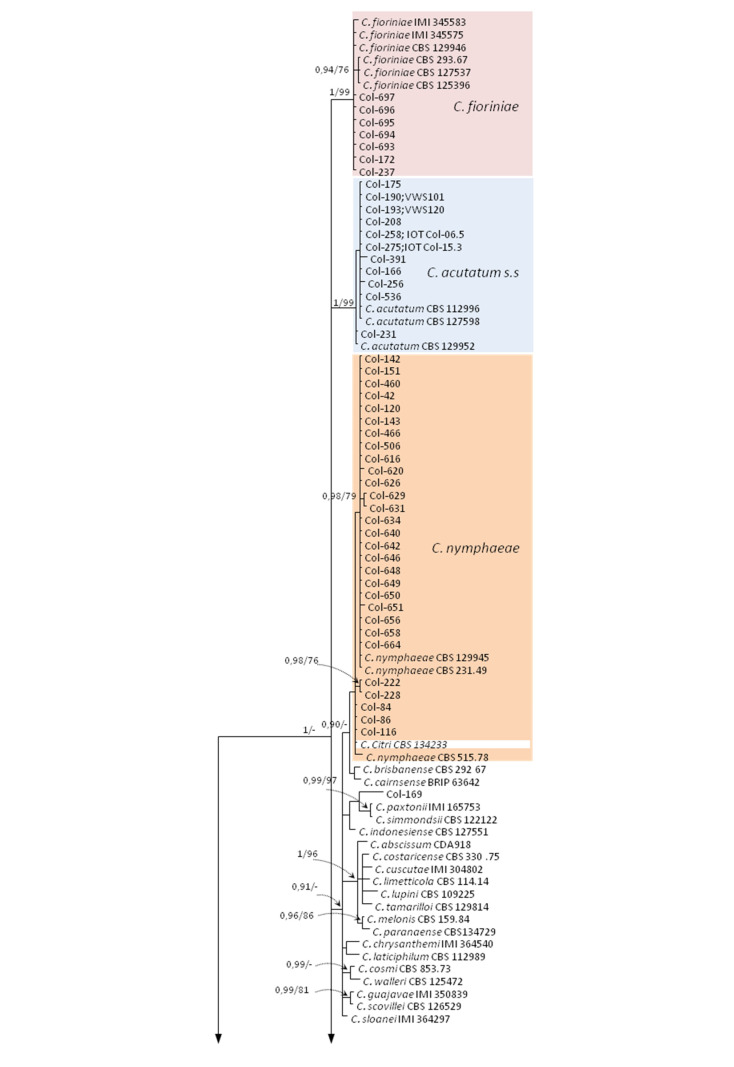

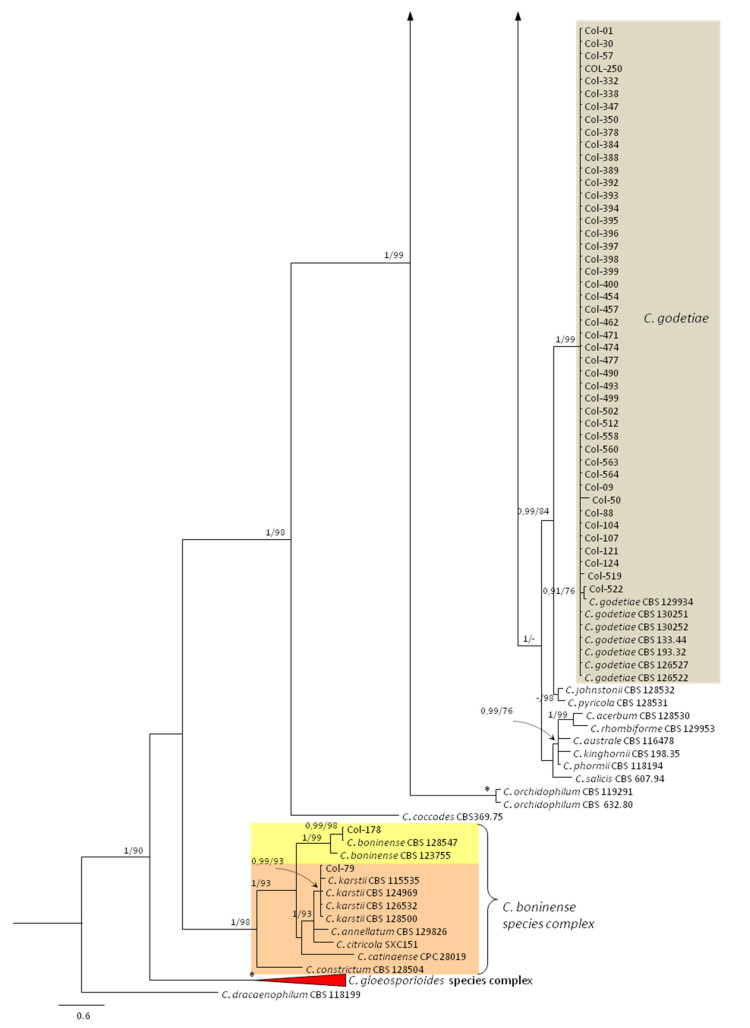
Phylogenetic tree resulting from Bayesian analysis using the combined ITS and TUB2 sequence alignments of *Colletotrichum acutatum*, *C. boninense*, and *C. gloeosporioides* species complexes. Bayesian posterior probabilities (PP, > 0.9) and bootstrap support values (MP, (BS) > 70%) of maximum parsimony analysis are shown in the nodes (PP/MP). The asterisk (*) indicates full support (1/100). *Colletotrichum dracaenophilum* (CBS 118199) was used as outgroup.

Our *Colletotrichum* isolates were initially identified based on the combined data of ITS and TUB2 sequences alignment. This first analysis (*dataset I*) included 186 taxa from which 109 were sequences of our isolates, and 77 were reference sequences from GenBank including the outgroup *C. dracaenophylum* isolate CBS 118199. A total of 867 characters, including gaps, were analyzed (ITS from 1 to 500, and TUB2 from 501 to 867 position). For BI analysis, a K2 + G model was used to combine both regions, and the phylogenetic tree is shown in Figure 3. In the MP analysis of the ITS and TUB2 regions, there were 811 positions in the final dataset, from which 220 characters were parsimony-informative and 591 conserved sites. The five most parsimonious trees were retained (TL = 555 steps, CI = 0.520, RI = 0.956, RC = 0.539, and HI = 0.480). The consensus tree obtained by MP analysis confirmed the topology obtained with BI, and bootstrap supports agreed with Bayesian probability values. Our isolates were grouped into three well-supported clades in this first phylogenetic tree according to the three *Colletotrichum* species complexes. Likewise, 93 isolates (from different countries and hosts) were grouped into the *C. acutatum* species complex, two isolates (Col-178 and Col-79, from Australia and Spain, and olive and sweet orange fruits, respectively) were grouped into the *C. boninense* species complex, and 14 isolates (from Australia, Spain and Tunisia, and most of them from olive trees) were grouped into the *C. gloeosporioides* species complex. These three different clades were well supported with a Bayesian posterior probability (PP) value of 1.0 for all of them, and with bootstrap support (MP (BS); %) values of 99%, 98%, and 100% for *C. acutatum*, *C. boninense*, and *C. gloeosporioides* species complexes, respectively. Most of the isolates belonging to the *C. acutatum* species complex clustered in four clades: (i) 45 isolates clustered together with reference isolates of *C. godetiae* (PP/BS(%):1/99), (ii) 11 isolates clustered with reference isolates of *C. acutatum* (1/99), (iii) 29 isolates clustered with reference isolates of *C. nymphaeae* (<0.90/70), and (iv) 7 isolates clustered with reference isolates of *C. fioriniae* (1/99). The isolate Col-169 from olive fruit (Australia) could not be well-identified with this phylogenetic analysis due to the fact that it clustered between reference sequences of *C. paxtonii* (IMI 165753) and *C. simmondsii* (CBS 122122) within the *C. acutatum* species complex.

Concerning the *C. boninense* species complex, the isolate Col-178 (from olive fruit, Australia) clustered together with reference isolates of *C. boninense* (1/99), and the isolate Col-79 (from sweet orange, Spain) clustered together with reference sequences of *C. karstii* (0.99/93). ITS and TUB2 multilocus alignment was not helpful to distinguish between species belonging to the *C. gloeosporioides* species complex, which formed a unique clade (1/100) (Figure 3).

**Figure 4:**
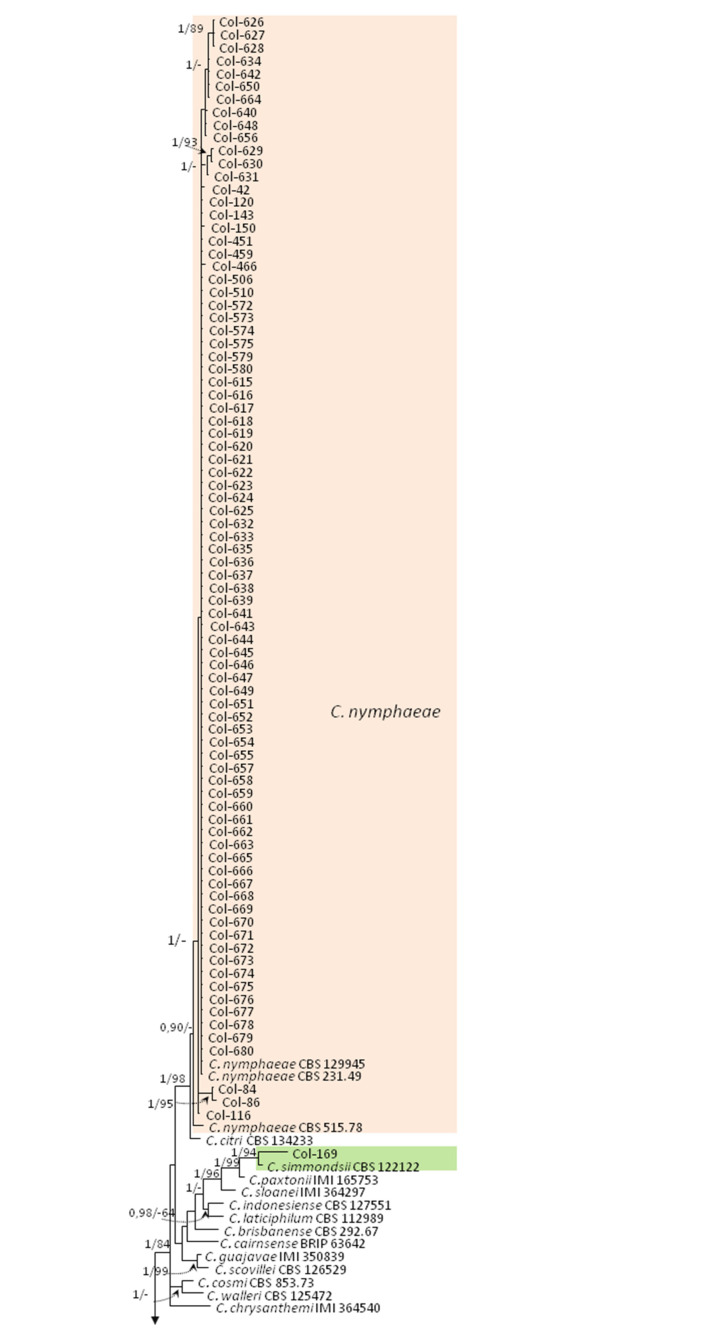

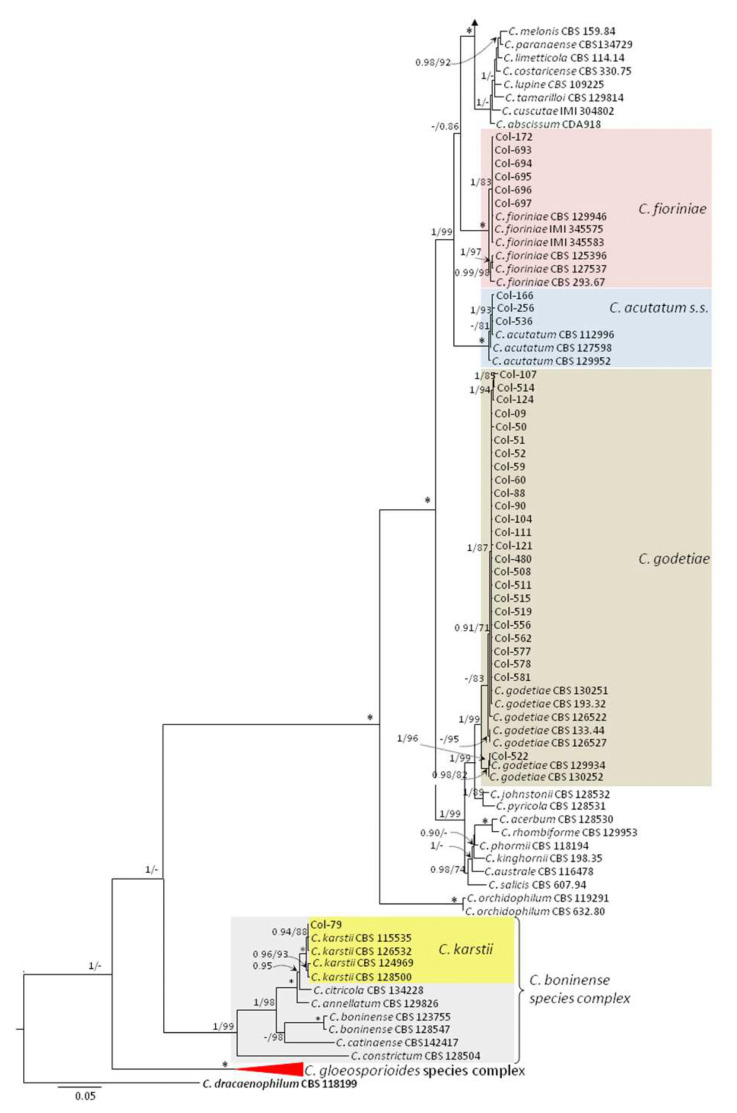
Phylogenetic tree obtained by Bayesian analysis using the combined ITS, TUB2, ACT, CHS-1, HIS3, and GAPDH sequence alignments of *Colletotrichum acutatum*, *C. boninense*, and *C. gloeosporioides* species complexes. Bayesian posterior probabilities (PP, > 0.9) and bootstrap support values (MP, (BS) > 70%) of maximum parsimony analysis are shown in the nodes (PP/MP). The asterisk (*) indicates full support (1/100). *Colletotrichum dracaenophilum* (CBS 118199) was used as outgroup.

A second multigene analysis (*dataset II*) was performed based on ITS, TUB2, ACT, CHS-1, HIS-3, and GADPH regions with a total of 188 taxa from which 126 were sequences of our isolates, and 62 were reference sequences from GenBank, including the outgroup *C. dracaenophylum* isolate CBS 118199. A total of 2143 characters, including gaps, were analyzed. The gene boundaries in the multialignment were ITS (from 1 to 518 positions), TUB2 (519–902), ACT (903–1186), CHS-1 (1187–1464), HIS-3 (1465–1853), and GADPH (1854–2143). For Bayesian analysis, a K2 + G model was selected for ITS, a K2 + I model for TUB2 and ACT, a TN93 + G model for CHS-1 and HIS-3, and a K2 + G+I model for GADPH, and they were incorporated in the analysis. The tree obtained with Bayesian PP values is shown in Figure 4.

Regarding MP analysis, there were 1845 positions in the final dataset, from which 693 characters were parsimony-informative, 1266 conserved sites, and 114 parsimony-uninformative. Two most parsimonious trees were retained (TL = 1673 steps, CI = 0.525, RI = 0.944, RC = 0.529, HI = 0.475). The consensus tree obtained by MP analysis confirmed the topology obtained with Bayesian inference, and BS values agreed with Bayesian probability values. This second phylogenetic improved the identification of isolates belonging to the *C. acutatum* species complex. Regarding our isolates, 119 were grouped as *C. acutatum* species complex, one isolate (Col-79) was grouped as *C. boninense* complex, and 14 isolates were grouped as *C. gloeosporioides* species complex. These three clades were well supported with a PP value of 1.0 and BS values of ≥ 99%. The isolates belonging to the *C. acutatum* species complex clustered in five well-supported clades: (i) 84 isolates (one from Italy, 73 from Portugal, and 10 from Spain, most of them from olive trees) clustered together with three reference isolates of *C. nymphaeae* (1/98), (ii) 25 isolates (20 from Spain, and 5 from Portugal, all of them from olive trees except Col-522 from almond trees) clustered with seven reference isolates of *C. godetiae* (1/99), (iii) 6 isolates from olive trees (five from California and one from Australia) clustered with six reference isolates of *C. fioriniae* (1/100), and (iv) 3 isolates with different origins (Australia, Tunisia, and Spain) clustered with the reference isolates of *C. acutatum* (1/100). In this case, the isolate Col-169 from olive trees (Australia), which could not be identified before based on ITS and TUB2-combined alignment, clustered consistently (1/94) with the reference ex-type isolate of *C. simmondsii* CBS 122122. The combined alignment of ITS, TUB2, ACT, CHS-1, HIS-3, and GADPH regions was insufficient to distinguish between species belonging to the *C. gloeosporioides* species complex (Figure 4).

**Figure 5:**
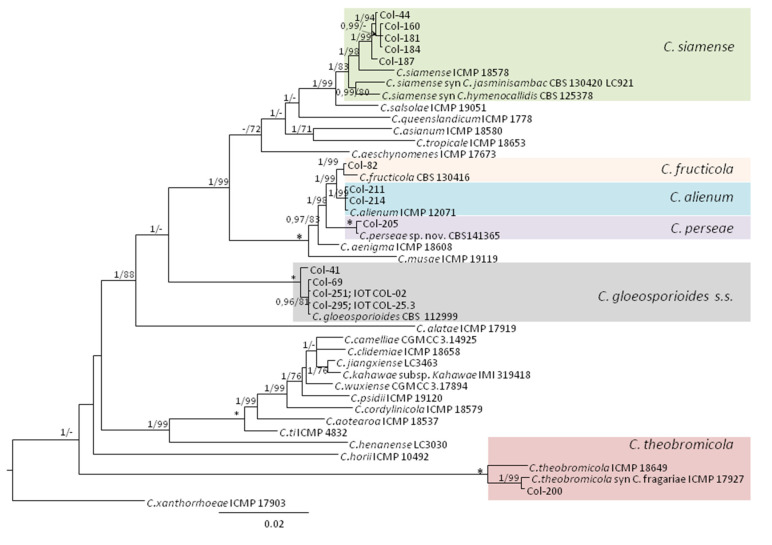
Phylogenetic tree obtained by Bayesian analysis using the combined ITS, TUB2, and ApMat sequence alignments of the *Colletotrichum gloeosporioides* species complex. Bayesian posterior probabilities (PP, > 0.9) and bootstrap support values (MP, (BS) > 70%) of Maximum Parsimony analysis are shown in the nodes (PP/MP). The asterisk (*) indicates full support (1/100). *Colletotrichum xanthorrhoeae* (ICMP 17903) was used as outgroup.

Finally, an additional multilocus alignment combining ITS, TUB2, and ApMat gene sequences (*dataset III*) was performed for inferring organismal phylogeny of the isolates belonging to the *C. gloeosporioides* species complex. It included 42 taxa, from which 14 were sequences of our isolates, and 29 were reference sequences from GenBank including the outgroup *C. xanthorrhoeae* ICMP17903. A total of 1749 characters, including gaps, were processed. The gene boundaries in the multialignment were ITS (1–484), TUB2 (485–842), and ApMat (843–1749). For Bayesian analysis, a K2 + G model was selected for ITS and ApMat, while a K2 model was used for TUB2. The tree obtained with Bayesian posterior probability values is shown in Figure 5. Regarding MP analysis, there were a total of 1576 positions in the final dataset, from which 363 characters were parsimony-informative, 1097 conserved sites, and 116 parsimony-uninformative. The four most parsimonious trees were retained (TL = 869 steps, CI = 0.764, RI = 0.894, RC = 0.683, and HI = 0.236). MP analysis confirmed the tree obtained by BI and BS agreed with PP values. The 14 isolates from this study classified within the *C. gloeosporioides* complex were identified as *C. alienum* (two isolates from olive trees, Australia; 1/99), *C. fructicola* (one isolate from olive trees, Spain; 1/99), *C. gloeosporioides* (four isolates, two of them from Tunisia (Col-251 and Col-295); and the other two from Spanish olive (Col-41) and sweet orange trees (Col-69); 1/100), *C. perseae* (one isolate from olive trees, Australia; 1/100), *C. siamense* (five isolates, four of them from olive trees Australia, and one from strawberry, Spain (Col-44; 1/83), and *C. theobromicola* syn. *C. fragariae* (one isolate from olive trees, Australia; 1/99) (Figure 5).

**Figure 6:**
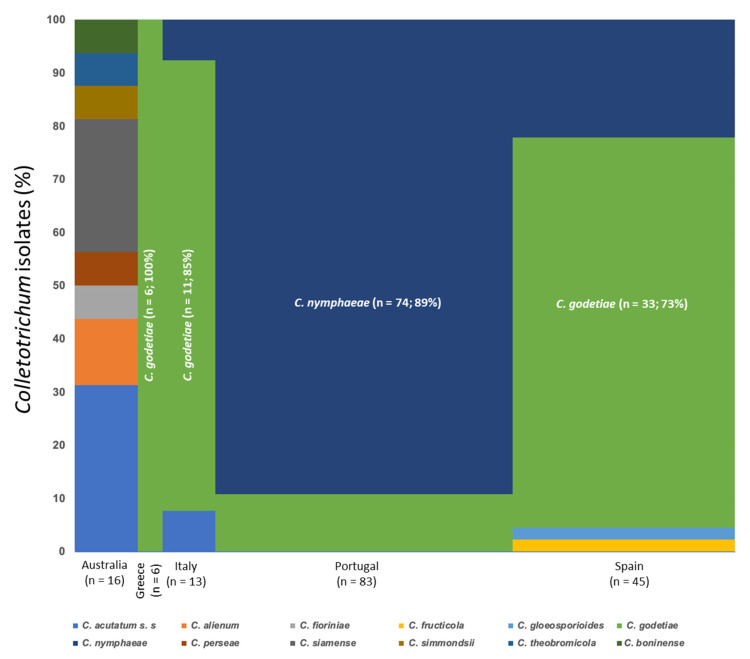
Mosaic Plot representing the relative percentage of twelve *Colletotrichum* species in the five countries where six or more fungal isolates were studied. *n* = number of *Colletotrichum* isolates analyzed.

Considering just the new olive tree isolates in this study, we molecularly identified 177 isolates belonging to 12 species of *Colletotrichum*. Most of these were Portuguese and Spanish isolates, 83 and 45 isolates, respectively. The species *C. nymphaeae* and *C. godetiae* were the most frequent (87 and 59 isolates, respectively) followed by *C. acutatum*, *C. fioriniae,* and *C. gloeosporioides* with 10, 7, and 3 isolates, respectively. The rest of the species (*C. boninense*, *C. fructicola*, *C. perseae*, *C. simmondsii*, and *C. theobromicola*) were represented by just one. At the same time, two and four Australian isolates were identified as *C. alienum* and *C. siamense,* respectively. Overall, *C. godetiae* was the dominant species in all the European countries (Greece, Italy, and Spain) except in Portugal, where the *C. nymphaeae* (89%) was the predominant species followed by *C. godetiae* (11%) (Figure 6).

Besides the isolates from olive trees, we have included eight *Colletotrichum* isolates obtained from other hosts in Spain, such as almonds, sweet orange trees, strawberries, and terebinth (*Pistacia terebinthus*). Among these isolates, six species were identified: *C. acutatum* from an almond tree, *C. godetiae* from almond and terebinth trees, *C. nymphaeae* (two isolates) and *C. siamense* from strawberries, and *C. gloeosporioides* and *C. karstii* from a sweet orange tree (Table 1).

### 3.4. Pathogenicity Tests

#### 3.4.1. Pathogenicity to Olive Fruit

**Figure 7:**
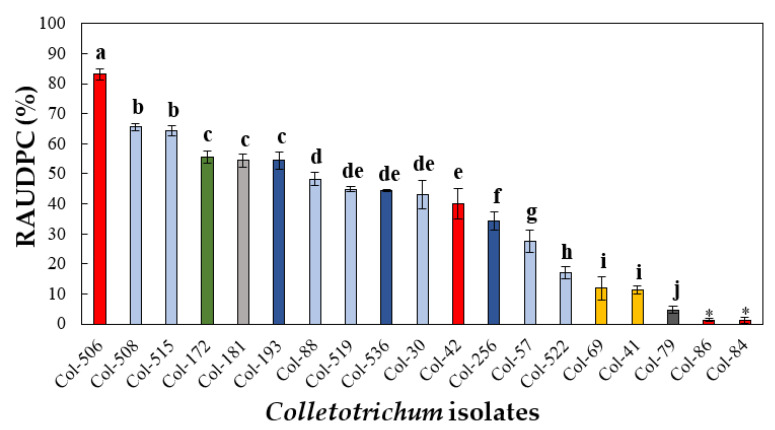
Relative area under the disease progression curve (RAUDPC) on the fruits of olive cv. Hojiblanca inoculated with the following isolates: *C. acutatum* (dark-blue columns) from olives (Col-193 and Col-256) and almonds (Col-536); *C. fioriniae* (green column) from olives (Col-172); *C. gloeosporioides* (yellow columns) from olives (Col-41) and sweet oranges (Col-69); *C. godetiae* (light-blue columns) from olives (Col-30, Col-57, Col-88, Col-508, Col-515, and Col-519) and almonds (Col-522); *C. kasrstii* (dark-gray column) from sweet oranges (Col-79), *C. nymphaeae* (red column) from olives (Col-42 and Col-506) and strawberries (Col-84 and Col-86), and *C. siamense* (light-gray column) from olives (Col-181). Columns are the means of two independent sets (experiments) of three replicated (humid chambers) with 20 fruits per humid chamber. Vertical bars are the standard error of the means. Columns with the same letter do not differ significantly according to Tukey’s HSD test at *p* = 0.05. * Isolates non-pathogenic to olives.

**Figure 8:**
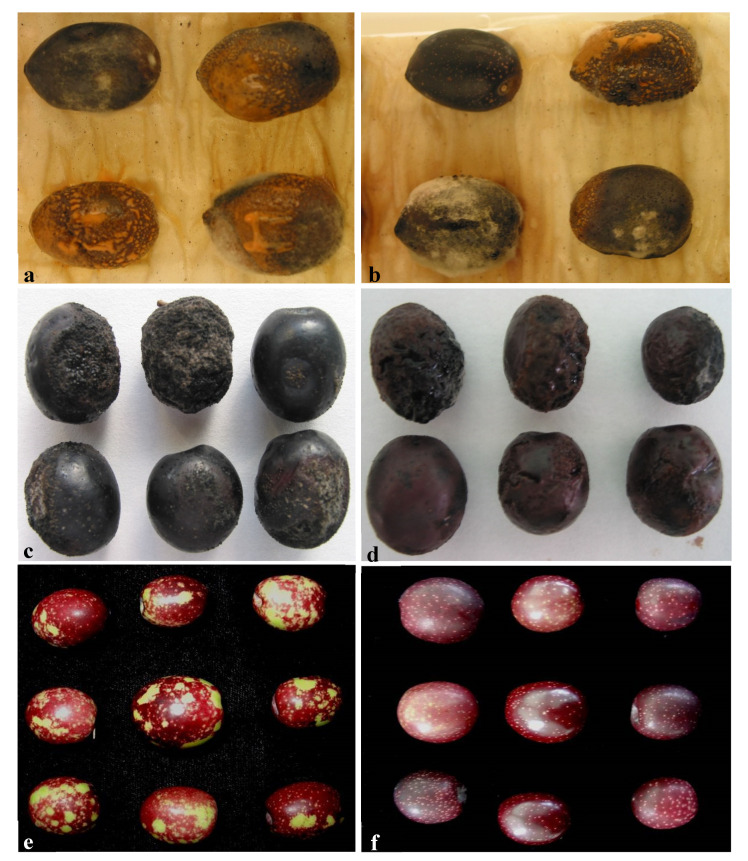
Anthracnose symptoms developed on non-wounded violet (color class 3) olive fruit of cv. Hojiblanca 14 days after inoculation with conidial suspension of the following isolates: (**a**), *Colletotrichum nymphaeae* from olives (Col-42); (**b**), *C. godetiae* from olives (Col-57); (**c**), *C. karstii* from sweet oranges (Col-79); (**d**), *C. gloeosporioides* from sweet oranges (Col-69); (**e**), *C. nymphaeae* from strawberries (Col-84) and (**f**), non-inoculated control olive fruit.

Significant differences in virulence (*p* ≤ 0.001) were observed between isolates depending on the *Colletotrichum* species and the original host. Most of the isolates tested were pathogenic in olive fruit except for *C. nymphaeae* isolates Col-84 and Col-86, and both originated from strawberries. Among the pathogenic isolates, RAUDPC values varied from 4.7% to 83.1% for *C. karstii* isolate Col-79 and *C. nymphaeae* isolate Col-506. The olive tree isolates Col-508 and Col-515 of *C. godetiae*, together with the isolates Col-506 of *C. nymphaeae*, were the most virulent olive fruit (RAUDPC > 64%) (Figure 7 and Figure 8). Overall, olive tree isolates belonging to *C. godetiae* and *C. nymphaeae* caused the typical “soapy rot”, i.e., rot covering the totality of the fruit surface with abundant conidia in mucilaginous orange masses.

#### 3.4.2. Pathogenicity on Other Hosts

**Figure 9:**
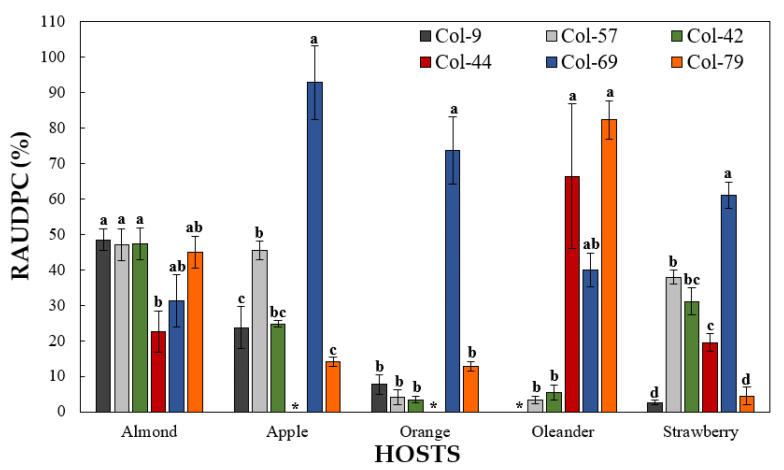
Relative area under the disease progression curve (RAUDPC) on fruits of almond cv. Guara, apple cv. Golden Delicious, sweet orange cv. Lane Late, strawberry cv. Camarosa, and on oleander leaves inoculated with the following isolates: *Colletotrichum godetiae* from olive (Col-9 and Col-57), *C. gloeosporioides* from sweet orange (Col-69), *C. karstii* from sweet oranges (Col-79), *C. nymphaeae* from olives (Col-42), and *C. siamense* from strawberries (Col-44). Columns are the means of two independent sets (experiments) of three replicated (humid chambers) in each host inoculation, with 10 fruit or leaves per host and per humid chamber. Vertical bars are the standard error of the means. For each host, columns with the same letter are not significantly different according to the Tukey’s HSD test at *p* = 0.05. * Isolates not pathogenic to these hosts.

**Figure 10:**
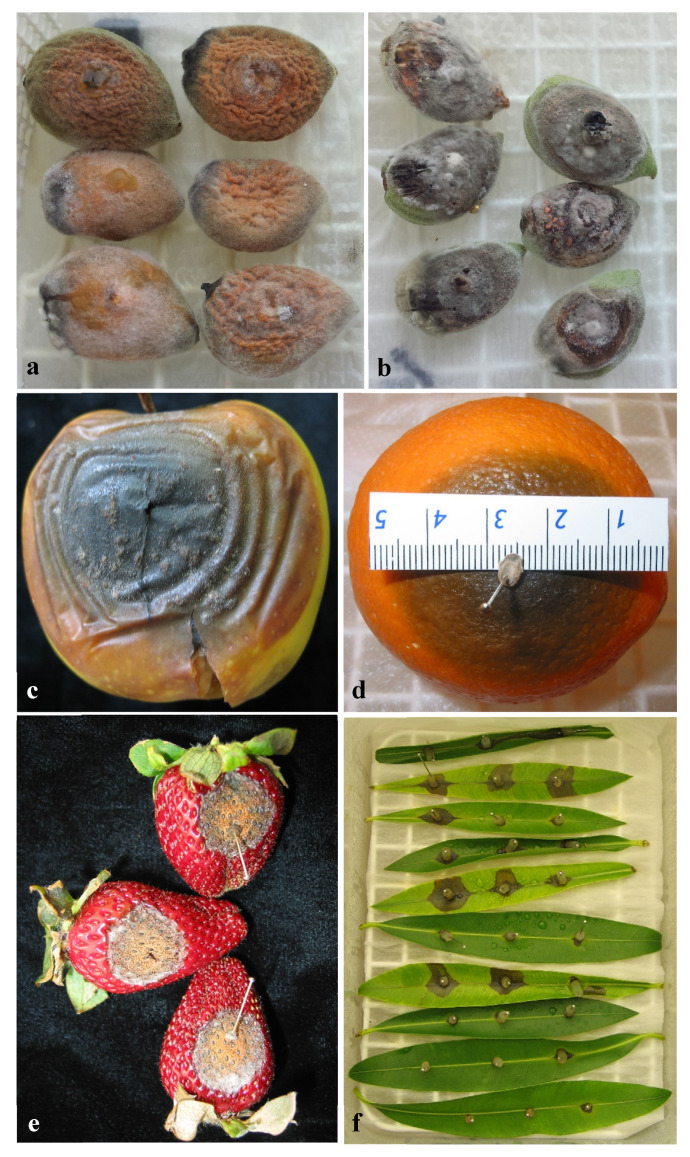
Anthracnose symptoms developed on fruit or leaves of several hosts 14 days after inoculation with a conidial suspension of *Colletrotrichum* isolates. (**a**,**b**) almond cv. Guara inoculated with *C. acutatum* from almonds (Col-536) and *C. godetiae* from almonds (Col-522) (**c**) apple cv. Golden Delicious inoculated with *C. gloeosporioides* from sweet oranges (Col-69); (**d**) sweet orange cv. Lane Late inoculated with *C. gloeosporioides* from sweet oranges (Col-69); (**e**) strawberry cv. Camarosa inoculated with *C. godetiae* from strawberries (Col-57); (**f**) leaves of *Nerium oleander* inoculated with *C. gloeosporioides* from sweet oranges (Col-69).

Most of the *Colletotrichum* isolates tested in this experiment were pathogenic in all the hosts evaluated except for *C. siamense* isolate Col-44 from strawberries, which was non-pathogenic to apple and sweet orange fruits, and *C. godetiae* isolate Col-9, which was non-pathogenic to oleander leaves. There was a significant interaction between isolate and host. In almond fruit, for example, all the *Colletotrichum* isolates were pathogenic with significant (*p* = 0.017) differences in virulence among them. In this host, *C. siamense* isolate Col-44 from strawberries was the less virulent (RAUDPC = 22.6%), while the isolates Col-9 and Col-57 (*C. godetiae*), and Col-42 (*C. nymphaeae*) caused RAUDPCs around 50%. In apple and sweet orange fruit, *C. gloeosporioides* isolate Col-69 from sweet oranges was the most virulent (RAUDPC > 70%) with marked differences in virulence concerning the other isolates tested. In both apples and sweet oranges, *C. siamense* isolate Col-44 was not pathogenic. Concerning pathogenicity on oleander leaves, *C. karstii* Col-79 was the most virulent isolate (RAUDPC = 82.3%) followed by *C. siamense* isolate Col-44 (RAUDPC = 66.1%). Conversely, *C. godetiae* isolate Col-57 and *C. nymphaeae* isolate Col-42 were weakly pathogenic (RAUDPC < 5.0%). Finally, in strawberry fruit, *C. gloeosporioides* Col-69 was the most virulent isolate (RAUDPC = 61.1%), while *C. godetiae* isolate Col-57, *C. nymphaeae* isolate Col-42, and *C. siamense* isolate Col-44 showed moderate levels of virulence (RAUDPC = 37.9%, 31.1%, and 19.4%, respectively). The isolates *C. godetiae* Col-9 and *C. karstii* Col-79 showed the lowest levels of virulence in strawberry fruit (RAUDPC < 5%) (Figure 9 and Figure 10).

## 4. Discussion

Fungal species belonging to the *Colletotrichum* genus are characterized by a global distribution associated with anthracnose diseases affecting a wide range of hosts, including many tree crops [5,58,59,60]. Although numerous species of *Colletotrichum* have been associated with the olive crop, these studies have focused on specific producing regions and lack an overall view [4,5,23,24,31]. Interestingly, the diversity of *Colletotrichum* species affecting olive trees in Spain, the leading olive oil producer globally, is very little known since the main study was conducted before dividing the *Colletotrichum* species complex into species to molecular profiles [33]. The present work focused on elucidating the biodiversity of the *Colletotrichum* species, causing olive anthracnose worldwide, emphasizing the fungi population from the Iberian Peninsula, i.e., Spain and Portugal. To this end, a vast collection of *Colletotrichum* isolates obtained mainly from olives in the main olive-growing regions of the world (Australia, Brazil, California, Greece, Italy, Portugal, Spain, Tunisia, and Uruguay) were characterized based on morphological, molecular, and pathogenic characters.

Several *Colletotrichum* species can produce infections in a single host, showing high pathogenic specialization; much more frequent, however, are the *Colletotrichum* species with the ability to infect multiple hosts [58,60]. These antecedents suggest that correct taxonomic identification of *Colletotrichum* species is essential to avoid etiological ambiguities. Therefore, determining the aetiology of *Colletotrichum* diseases will be crucial to develop studies on the epidemiology and control of the disease in the future [10].

By tradition, the taxonomic identification of the species of *Colletotrichum* genus has been mainly based on phenotypic differences of colony morphology and conidium shape and size [59,61,62]. Several authors considered the curvature of the ends of the conidia as the essential morphological character to distinguish between *Colletotrichum* species [16,62,63]. This conidial character has been traditionally used to discriminate between *C. acutatum* (sharp conidium ends, fusiform) and *C. gloeosporioides* (rounded conidium ends, ellipsoid) [58,64]. Nevertheless, conidia of the isolates characterized morphologically in this study varied in form (ellipsoid, clavate, or fusiform) between fungal isolates within the same species complexes and, even, the same fungal species. For example, within the *C. acutatum* species complex, the three shapes of conidia were observed for *C. acutatum* isolates, whereas *C. godetiae* isolates showed clavate conidia except for the isolates Col-88 and Col-522, which showed ellipsoid and fusiform conidia, respectively. Because of this morphological characteristic (clavate conidia), Faedda et al. [26] described the new species *C. clavatum* as the most common associated with olive anthracnose in Italy. However, this new species did not show molecular and morphological differences with the previous one, *C. godetiae* [27]. Likewise, in this study, and previous ones, different types of conidia were also observed between species belonging to *C. boninense* species complex (clavate or ellipsoid conidia) as well as within the *C. gloeosporioides* species complex (ellipsoid or clavate conidia).

Regarding the colony color, there were no differences that allowed their specific identification. Usually, colonies of *C. godetiae* isolates were gray, *C. acutatum* isolates showed pink tones, and *C. fioriniae* and *C. gloeosporioides* isolates showed light gray ones. The only exception was *C. siamense* from strawberries (Col-44), which showed a distinctive greenish-gray colony color. Similar results about differences between *Colletotrichum* species affecting almond trees depending on colony color-subpopulations were described by López-Moral et al. [36], who indicated that *C. acutatum, C. godetiae,* and *C. nymphaeae* isolates were associated with pinkish-orange, dark gray, and light gray subpopulations, respectively. Despite these specific differences in colony color between *Colletotrichum* species, it does not correctly identify *Colletotrichum* species since environmental factors could significantly influence the stability of morphological traits becoming in intermediate forms [58,65,66].

Regarding the sensitivity to the benomyl, all the isolates included within *C. boninense* and *C. gloeosporioides* species complexes were highly sensitive to the fungicide with inhibition percentages of mycelial growth higher than 93%. Conversely, the isolates belonging to the *C. acutatum* species complex were more tolerant (from 33.5% to 71.1%) to the fungicide than those of the *C. gloeosporioides* species complex. However, there was an unclear association between fungicide tolerance and pathogen species or geographic or host origin. Our results are concordant with those obtained by several authors who indicated that *C. gloeosporioides* isolates are highly sensitive to benomyl while *C. acutatum* isolates are moderately tolerant independent of the host origin [4,43,58,67,68]. Species belonging to the *C. acutatum* complex are predominant in the olive-growing areas in the Andalusia region [7]. Thus, Andalusian *C. acutatum* isolates show higher tolerance to benomyl than those of *C. gloeosporioides* and *C. boninense*. These differences could be a consequence of the use of this fungicide in olive orchards. However, benomyl had not been traditionally used by olive growers in Andalusia to prevent olive diseases and is currently not registered for use [7].

The ability of *Colletotrichum* isolates to hydrolyze casein was also helpful to discriminate isolates between *Colletotrichum* species complexes, but with some exceptions. Thus, most isolates belonging to the *C. acutatum* species complex hydrolyzed the casein, whereas those belonging to *C. boninense* and *C. gloeosporioides* species complexes did not. These results are in concordance with those obtained by Martín et al. [69].

Molecular techniques, such as phylogenetic analyses of ribosomal genes (i.e., ITS, 28S, etc.) and functional protein regions (i.e., actin, β-tubulin, calmodulin, etc.) have been set up during these last few years, improving the identification of *Colletotrichum* species within this genus [27,50,54,66,70]. The combined alignment of ITS and TUB2 helped identify the isolates into the three *Colletotrichum* species complexes: *C. acutatum*, *C. boninense*, and *C. gloeosporioides*. In general, ITS and TUB2 were enough to infer *Colletotrichum* species within *C. acutatum* and *C. boninense* complexes except for the isolate Col-169, which was identified as *C. simmondsii* based on the ITS, TUB2, ACT, CHS-1, HIS-3, and GADPH regions.

In corroboration with previous studies [54,70,71,72], we used an additional alignment combining ITS, TUB2, and ApMat gene sequences for inferring the phylogeny of the isolates previously grouped as *C. gloeosporioides* species complex. Phylogenetic studies conducted to determine the provided information by ApMat and glutamine synthetase (GS) showed that regions offer similar information, but ApMat discriminates more species in the *C. gloeosporioides* species complex [70,72].

All the aspects discussed above are in agreement with the ideal polyphasic approach for *Colletotrichum* systematics described by Cai et al. [73], who suggested that the identification of *Colletotrichum* species should be based on multi-gene phylogenetic analysis together with recognizable phenotypic characters, such as morphology, physiology, pathogenicity, or cultural characteristics, among others.

Concerning the global distribution of our *Colletotrichum* isolates, most of those collected from olive trees in Spain were classified within the *C. acutatum* species complex, with *C. godetiae* being the most common species, followed by *C. nymphaeae*. The Spanish isolates of *C. godetiae* were collected in the Andalusia region, whereas *C. nymphaeae* isolates showed more diversity regarding the country’s geographic origin. We previously observed that olive anthracnose is caused by the *C. acutatum* species complex in the olive-growing areas of southern Spain [7]. Still, the molecular identification of these isolates has not been conducted until the present study. The species *C. gloeosporioides* and *C. fructicola* were also isolated from olive trees in Valencia (Eastern Spain) and Catalonia (North-Eastern Spain). Remarkably, the species *C. fructicola* (Col-82) was isolated from olive leaves showing necrotic lesions (an unusual symptom for anthracnose) from plants in a nursery, probably due to cross-contamination with citrus plants. The infection/contamination of olive stock with *Colletotrichum* could influence the long-distance spread of these pathogens.

In our study, most olive isolates from Greece, Italy, and Tunisia were identified as *C. godetiae*. These results are in concordance with those obtained by several authors who indicate that the species belonging to the *C. acutatum* complex are considered the most important ones associated with olive anthracnose in European countries [6,24,26,32]. Conversely, and in concordance to the previous studies [4,25], our study confirmed that the most prevalent species associated with olive anthracnose in Portugal is *C. nymphaeae*. In initial studies, we observed that the Spanish *C. godetiae* isolates, coming from olive-growing regions where copper-based fungicides are frequently used by farmers, are more tolerant to copper than *C. nymphaeae* isolates, while in Portugal, the opposite is true. However, the adaptation to weather and agronomic conditions, including the potential specialization in the local olive cultivars, could explain these differences [7,74]. In addition, in previous studies we occasionally detected interactions between olive cultivar-*Colletotrichum* spp., but none so important so as to explain such a different species distribution [7,8,9,57].

Although we identified eight *Colletotrichum* species among the Australian isolates, neither *C. godetiae* nor *C. nymphaeae* were found. This substantial variability of species associated with olive anthracnose in Australia was influenced by the fact that the 16 studied isolates were previously selected from a higher search to maximize the variability. Besides, previous studies hypothesized that the center of origin of *Colletotrichum* could be in Oceania since the highest level of variability and strains of the species complexes occurred mainly in Australia and New Zealand [59]. The species *C. siamense* and *C. theobromicola* have been previously described in olive trees in Australia [23]. However, the species *C. perseae* (Col-205) was identified for the first time as associated with olive anthracnose. The species *C. alienum* has been identified in a broad diversity of hosts, including olive trees [13,53,75], while *C. perseae* has been described as novel species associated with avocado anthracnose in Israel [21].

All of the isolates from olive trees from California were identified as *C. fioriniae*. Nevertheless, the etiological studies of olive anthracnose in this state have not been conducted yet. However, *C. fioriniae* is a common pathogen of nut trees in California [27,76].

Finally, among all our isolates, species belonging to the *C. gloeosporioides* complex were only identified for the isolates collected from Australia, Tunisia, and Eastern Spain. These results agree with those described by Talhinhas et al. [5], who indicated that the *C. gloeosporioides* complex occurs in several countries presenting lower frequency than other species. On the other hand, these authors described *C. acutatum* as the prevalent species complex associated with olive anthracnose in the Southern Hemisphere.

Regarding the six *Colletotrichum* species obtained from hosts other than the olive tree (i.e., *C. acutatum, C. gloeosporioides*, *C. godetiae*, *C. karstii*, *C. nymphaeae* and *C. siamense*), it is interesting to note that all of them are new reports from the respective hosts in Spain, except *C. gloeosporioides* from sweet orange and *C. acutatum* from almond trees [36].

Among the *Colletotrichum* isolates from almonds, olives, sweet oranges, and strawberries tested for pathogenicity on olive fruit, only the isolates from strawberries were not pathogenic. Overall, the isolates from olive trees were more virulent in olive fruit than those from other hosts [35]. These results agree with López-Moral et al. [36], who observed that *Colletotrichum* isolates from olives (Col-506 and Col-508) were more virulent than ones from almonds (Col-522 and Col-536) on olive fruit. Overall, the pathogenicity in olive fruit has been confirmed in eight species, which differ in their virulence [5,35]. These pathogenicity tests have demonstrated that *C. acutatum* and *C. nymphaeae* are the most virulent species, *C. godeatiae* and *C. fioriniae* resulted in an intermediate virulence, and *C. gloeosporioides* is less virulent [4,23,24]. When cross inoculations were conducted using different isolates and hosts, a notable pathogenic specialization was observed in some cases. For example, *C. siamense* isolate Col-44 from strawberries resulted as non-pathogenic to apple and sweet orange fruit [7]. Although we can find many differences in virulence between isolates and host combinations, our results demonstrated the pathogenic specialization of *Colletotrichum* isolates on their host. This characteristic has been used to identify specific or intraspecific taxa in this genus [16,61]. However, further research is needed to determine the pathogenic specialization of *Colletotrichum* isolates on olive trees.

In conclusion, in the present study, the largest so far, we recorded 12 species of the pathogen affecting the olive tree, *C. acutatum*, *C. alienum*, *C. boninense*, *C. fioriniae*, *C. fructicola*, *C. gloeosporioides*, *C. godetiae*, *C. nymphaeae*, *C. perseae*, *C. siamense*, *C. simmondsii*, and *C. theobromicola*. According to our knowledge, this study is the first report of *C. boninense*, *C. fructicola*, and *C. perseae* affecting olive trees. Other studies have described another six *Colletotrichum* species associated with this crop, C. *aenigma*, *C. cigarro*, *C. karstii*, *C. queenslandicum*, *C. lupini*, and *C. rhombiborme* [4,5,23,77,78]. Although many other woody crops are affected by numerous species of *Colletotrichum* [21,54,79,80], the olive tree is one of the plant species affected by the most remarkable diversity of taxa of this fungal genus with 18 species. This fact may be associated with the enormous expansion capacity of the olive tree in the last 30 years, which has led it to be the main woody crop in the world [1]. Our results also showed that the dominant species in Spain, Italy, and Greece is *C. godetiae*, while *C. nymphaeae* is the dominant species in Portugal. Interestingly, neither of these two species have been described in Australia, where we have found the highest diversity with eight *Colletotrichum* spp. These results reinforce the hypothesis that native species of *Colletotrichum* to each place jumped from other hosts to the olive tree when it colonized new growing areas, rather than the pathogen having moved with the crop.

## 5. Conclusions

This study aimed to elucidate the biodiversity of *Colletotrichum* species causing olive anthracnose worldwide. Our results demonstrated that the phenotypic characters (colony and conidium morphology, benomyl-sensitivity, and casein-hydrolyse ability) are not helpful enough to identify *Colletotrichum* species, although they allow for the separation of some species complexes. For instance, conidia of the *Colletotrichum* isolates characterized morphologically in this study varied in form (ellipsoid, clavate, or fusiform) among fungal isolates within the same species complexes and even the same fungal species. Thus, molecular tools are essential to infer phylogenetic species within the *Colletotrichum* genus. In this respect, ITS and TUB2 are enough to distinguish *Colletotrichum* species within the *C. acutatum* and *C. boninense* species complexes. In contrast, ITS, TUB2, ACT, CHS-1, HIS-3, and GADPH regions were necessary to discriminate within the *C. gloeosporioides* complex. Consequently, our results reinforce the hypothesis based on the ideal polyphasic approach for *Colletotrichum* systematics, suggesting that the identification of *Colletotrichum* species should be based on multi-gene phylogenetic analysis together with recognizable phenotypic characters. Pathogenicity tests in olive showed significant differences in virulence to this host between isolates depending on the *Colletotrichum* species and host origin. When cross-pathogenicity was conducted, most of the *Colletotrichum* isolates tested were pathogenic in all the hosts evaluated, except for *C. siamense* to apple and sweet orange fruits, and *C. godetiae* to oleander leaves. Finally, regarding the diversity of *Colletotrichum* species causing olive anthracnose worldwide, among the 177 *Colletotrichum* isolates from olive included in this study, 12 *Colletotrichum* species belonging to *C. acutatum*, *C. boninense,* and *C*. *gloeosporioides* complexes were identified. The species *C. godetiae* was dominant in Spain, Italy, and Greece. The highest diversity was in Australia, where eight *Colletotrichum* species were identified. Altogether, this study also reinforces the hypothesis that native species of *Colletotrichum* to each place jumped from other hosts to the olive tree when it colonized new growing areas, rather than the pathogen having moved with the crop.

## Figures and Tables

**Table 1:** Isolates of *Colletotrichum* spp. used in this study, with collection details and GenBank accessions.

Species	Isolate ^b,c^	Origin, Year	Substrate, Host	GenBank Accession No. ^a^						
				ITS	TUB2	ACT	CHS-1	HIS3	GAPDH	ApMat
*C. abscissum*	**COAD 1877^T^**	Brazil, Cafelandia	*Psidium guajava*	KP843126	KP843135	KP843141	KP843132	KP843138	KP843129	-
*C. acerbum*	**CBS 128530^T^, ICMP 12921, PRJ 1199.3**	New Zealand	*Malus domestica*	JQ948459	JQ950110	JQ949780	JQ949120	JQ949450	JQ948790	-
*C. acutatum*	Col-166; UWS-65 ^d,e^	Australia; 2009	Fruit, *Olea europaea*	MH685231	MH713165	MH717594	MH801883	MH713299	MH717458	-
	Col-175; UWS-79 ^d,e^	Australia; 2009	Fruit, *Olea europaea*	MH685234	MH713166	-	-	-	-	-
	Col-190; UWS-101	Australia; 2009	Fruit, *Olea europaea*	MH685239	MH713167	-	-	-	-	-
	Col-193; UWS-120 ^f^	Australia; 2009	Fruit, *Olea europaea*	MH685240	MH713168	-	-	-	-	-
	Col-208;UWS-149 ^d,e^	Australia; 2009	Fruit, *Olea europaea*	MH685243	MH713169	-	-	-	-	-
	Col-231	Uruguay; 2010	Fruit, *Olea europaea* cv. Hojiblanca	MH685249	MH713170	-	-	-	-	-
	Col-256; IOT COL-04.1 ^f^	Nabeul, Tunisia; 2010	Fruit, *Olea europea* cv. Meski	KM594095	KP197006	MH717595	MH801884	MH713300	MH717459	-
	Col-258; IOT COL-06.5	Takelsa, Tunisia; 2010	Fruit, *Olea europea* cv. Arbequina	KM594093	KP185116	-	-	-	-	-
	Col-275; IOT COL-15.3	Nabeul, Tunisia; 2010	Fruit, *Olea europea* cv. Queslati	KM594101	KP197011	-	-	-	-	-
	Col-391	Bari, Italy; 2012	Fruit, *Olea europea* cv. Arbequina	MH685260	MH713171	-	-	-	-	-
	Col-536 ^d,f^	Lebrija, Sevilla, Spain; 2014	Fruit, *Prunus dulcis*	KY171894	KY171902	KY171910	KY171918	KY171926	KY171934	-
	**CBS 112996, ATCC 56816, STE-U 5292^T^**	Australia	*Carica papaya*	JQ005776	JQ005860	JQ005839	JQ005797	JQ005818	JQ948677	-
	**CBS 129952, PT227, RB015**	Portugal	*Olea europaea*	JQ948364	JQ950015	JQ949685	JQ949025	JQ949355	JQ948695	-
	**CBS 127598, 223/09**	South Africa	*Olea europaea*	JQ948363	JQ950014	JQ949684	JQ949024	JQ949354	JQ948694	-
*C. aenigma*	**ICMP 18608^T^**	Israel	*Persea americana*	JX010244	JX010389	-	-	-	-	KM360143
*C. aeschynomenes*	**ICMP 17673^T^**	USA	*Aeschynomene virginica*	JX010176	JX010392	-	-	-	-	KM360145
*C. alatae*	**ICMP 17919**	India	*Dioscorea alata*	JX010190	JX010383	-	-	-	-	KC888932
*C. alienum*	Col-211;UWS-152 ^d,e^	Australia; 2009	Fruit, *Olea europaea*	MH685244	MH713162	-	-	-	-	MH717580
	Col-214;UWS-156 ^d,e^	Australia; 2009	Fruit, *Olea europaea*	MH685245	MH713163	-	-	-	-	MH717581
	**ICMP 12071^T^**	New Zealand	*Malus domestica*	JX010251	JX010411	-	-	-	-	KM360144
*C. annellatum*	**CBS 129826^T^**	Colombia	Leaf, *Hevea brasiliensis*	JQ0055222	JQ005656	JQ005570	JQ005396	JQ005483	JQ005309	-
*C. aotearoa*	**ICMP 18537^T^**	New Zealand	*Coprosma* sp.	JX010205	JX010420	-	-	-	-	KC888930
*C. asianum*	**ICMP 18580^T^;** **CBS 130418**	Thailand	*Coffea arabica*	FJ972612	JX010406	-	-	-	-	FR718814
*C. australe*	**CBS 116478^T^**	South Africa	*Trachycarpus fortunei*	JQ948455	JQ950106	JQ949776	JQ949116	JQ949446	JQ948786	
*C. boninense*	Col-178; UWS-82 ^d,e^	Australia; 2009	Fruit, *Olea europaea*	MH685235	MH713152	-	-	-	-	-
	**CBS 123755^T^*,*** **MAFF 305972**	Japan	*Crinum asiaticum* cv. *sinicum*	JQ005153	JQ005588	JQ005501	JQ005327	JQ005414	JQ005240	-
	**CBS 128547, ICMP 10338**	New Zealand	*Camellia* sp.	JQ005159	JQ005593	JQ005507	JQ005333	JQ005420	JQ005246	-
*C. brisbanense*	**CBS 292.67^T^**	Australia	*Capsicum annuum*	JQ948291	JQ949942	JQ949612	JQ948952	JQ949282	JQ948621	
*C. cairnsense*	**BRIP 63642^T^, CBS 140847**	Australia	*Capsicum annuum*	KU923672	KU923688	KU923716	KU923710	KU923722	KU923704	-
*C. catinaense*	**CBS 142417^T^; CPC 27978**	Italy, Catania	*Citrus reticulata*	KY856400	KY856482	KY855971	KY856136	KY856307	KY856224	-
*C. chrysanthemi*	**IMI 364540, CPC 18930**	China	*Chrysanthemun coronarium*	JQ948273	JQ949924	JQ949594	JQ948934	JQ949264	JQ948603	-
*C. citri*	**CBS 134233**	China	*Citrus aurantiifolia*	KC293581	KC293661	KY855973	KY856138	KY856309	KC293741	-
*C. citricola*	**CBS 134228**	China	*Citrus unchiu*	KC293576	KC293656	KC293616	KY856140	KY856311	KC293736	-
*C. clidemiae*	**ICMP 18658^T^**	Hawaii, USA	*Clidemia hirta*	JX010265	JX010438	-	-	-	-	KC888929
*C. coccodes*	**CBS 369.75^T^**	The Netherlands	*Solanum tuberosum*	HM171679	JX546873	-	-	-	-	-
*C. constrictum*	**CBS 128504**	New Zealand	*Citrus limon*	JQ005238	JQ005672	JQ005586	JQ005412	KY856313	JQ005325	-
*C. cordylinicola*	**ICMP 18579^T^**	Thailand	*Cordyline fruticosa*	JX010226	JX010440	-	-	-	-	JQ899274
*C. cosmi*	**CBS 853.73, PD 73/856^T^**	The Netherlands	*Cosmos* sp.	JQ948274	JQ949925	JQ949595	JQ948935	JQ949265	JQ948604	-
*C. costaricense*	**CBS 330.75^T^**	Costa Rica	*Coffea arabica*	JQ948180	JQ949831	JQ949501	JQ949120	JQ949450	JQ948790	-
*C. cuscutae*	**IMI 304802, CPC 18873^T^**	Dominica	*Cuscuta* sp.	JQ948195	JQ949846	JQ949516	JQ949025	JQ949355	JQ948695	-
*C. dracaenophilum*	**CBS 118199**	China	*Dracaena*	JX519222	JX519247	JX519238	JX519230	JX546756	JX546707	-
*C. fioriniae*	Col-172;UWS-70 ^d,e,f^	Australia; 2009	Fruit, *Olea europaea*	MH685233	MH713172	MH717596	MH801885	MH713301	MH717460	-
	Col-237	Uruguay; 2010	Fruit, *Olea europaea* cv. Arbequina	MH685250	MH801882	-	-	-	-	-
	Col-693 ^d^	California, USA; 2017	Fruit, *Olea europaea*	MH685372	MH713173	MH717597	MH801886	MH713302	MH717461	-
	Col-694 ^d^	California, USA; 2017	Fruit, *Olea europaea*	MH685373	MH713174	MH717598	MH801887	MH713303	MH717462	-
	Col-695 ^d^	California, USA; 2017	Fruit, *Olea europaea*	MH685374	MH713175	MH717599	MH801888	MH713304	MH717463	-
	Col-696 ^d^	California, USA; 2017	Fruit, *Olea europaea*	MH685375	MH713176	MH717600	MH801889	MH713305	MH717464	-
	Col-697 ^d^	California, USA; 2017	Fruit, *Olea europaea*	MH685376	MH713177	MH717601	MH801890	MH713306	MH717465	-
	**IMI 345583, CPC 18889**	USA	*Fragaria × ananassa*	JQ948333	JQ949984	JQ949654	JQ005797	JQ005818	JQ948677	-
	**IMI 345575, CPC 18888**	USA	*Fragaria × ananassa*	JQ948332	JQ949983	JQ949653	JQ949116	JQ949446	JQ948786	-
	**CBS 125396; GJS 08-140A**	USA	*Malus domestica*	JQ948299	JQ949950	JQ949620	JQ948952	JQ949282	JQ948621	-
	**CBS 129946, PT170, RB021**	Portugal	*Olea europaea*	JQ948342	JQ949993	JQ949663	JQ949024	JQ949354	JQ948694	-
	**CBS 293.67, DPI 13120**	Australia	*Persea americana*	JQ948310	JQ949961	JQ949631	JQ948934	JQ949264	JQ948603	-
	**CBS 127537, STE-U 5289**	USA	*Vaccinium* sp.	JQ948318	JQ949969	JQ949639	JQ948935	JQ949265	JQ948604	-
*C. fructicola*	Col-82	Valencia, Spain; 2003	Leaf, *Olea europaea*	MH685214	MH713153	MH713292	MH713285	MH713414	MH717489	MH717582
	**CBS 130416, ICMP 18581**	Thailand	*Coffea arabica*	JX010165	JX010405	-	-	-	-	JQ807838
*C. gloeosporioides*	Col-41 ^d,e,f^	Montsia, Tarragona, Spain; 1999	Fruit, *Olea europaea*	MH685203	MH713154	MH713293	MH713286	MH713415	MH717490	MH717583
	Col-69 ^d,e,f^	Fuente la Palomera, Córdoba, Spain; 2001	*Citrus sinensis*	MH685212	MH713155	MH713294	MH713287	MH713416	MH717491	MH717584
	Col-251; IOT COL-02	Nabeul, Tunisia; 2010	Fruit, *Olea europaea*	KM594085	KP176441	-	-	-	-	MH717585
	Col-295; IOT COL-25.3	Nabeul, Tunisia; 2010	Fruit, *Olea europea* cv. Meski	KM594112	KP197021	-	-	-	-	MH717586
	**CBS 112999**	Italy	*Citrus sinensis*	JQ005152	JQ005587	JQ005500	JQ005326	JQ005413	JQ005239	JQ807843
*C. godetiae*	Col-1 ^d^	Almodóvar, Córdoba, Spain; 1998	Fruit, *Olea europaea* cv. Hojiblanca	MH685200	MH713003	-	-	-	-	-
	Col-9 ^d,e,f^	Antequera, Málaga, Spain: 1998	Fruit, *Olea europea* cv. Hojiblanca	MH685201	MH713004	MH717602	MH713178	MH713307	MH717466	-
	Col-30 ^d,e,f^	Llanos D. Juan, Córdoba, Spain; 1998	Fruit, *Olea europaea* cv. Hojiblanca	MH685202	MH713005	-	-	-	-	-
	Col-50 ^d,e^	Lucena, Córdoba, Spain, 1999	Fruit, *Olea europea*	MH685206	MH713006	MH717603	MH713179	MH713308	MH717467	-
	Col-51 ^d,e^	Lucena, Córdoba, Spain, 1999	Fruit, *Olea europea*	MH685207	MH713007	MH717604	MH713180	MH713309	MH717468	-
	Col-52 ^d^	Antequera, Málaga, Spain; 1999	Fruit, *Olea europea*	MH685208	MH713008	MH717605	MH713181	MH713310	MH717469	-
	Col-57 ^d,e,f^	Archidona, Málaga, Spain; 2002	Fruit, *Olea europaea*	MH685209	MH713009	-	-	-	-	-
	Col-59 ^d,e^	Archidona, Málaga, Spain; 2001	Fruit, *Olea europaea* cv. Hojiblanca	MH685210	MH713010	MH717606	MH713182	MH713311	MH717470	-
	Col-60 ^d,e^	Archidona, Málaga, Spain; 2001	Fruit, *Olea europaea* cv. Hojiblanca	MH685211	MH713011	MH717607	MH713183	MH713312	MH717471	-
	Col-88 ^d,e^	Montilla, Córdoba Spain; 2004	Fruit, *Olea europaea* cv. Picudo	MH685217	MH713012	MH717608	MH713184	MH713313	MH717472	-
	Col-90	CIFA Cabra, Córdoba Spain; 2004	Fruit, *Olea europaea* cv. Picudo	MH685218	MH713013	MH717609	MH713185	MH713314	MH717473	-
	Col-104	Cabra, Córdoba, Spain; 2014	Fruit, *Olea europaea* cv. Picudo	MH685219	MH713014	MH717610	MH713186	MH713315	MH717474	-
	Col-107	La Rambla, Córdoba, Spain;	Fruit, *Olea europaea* cv. Hojiblanca	MH685220	MH713015	MH717611	MH713187	MH713316	MH717475	-
	Col-111	Mengíbar, Jaén, Spain; 2014	Fruit, *Olea europaea* cv. Ocal	MH685221	MH713016	MH717612	MH713188	MH713317	MH717476	-
	Col-121	Montilla, Córdoba, Spain; 2014	Fruit, *Olea europaea* cv. Hojiblanca	MH685224	MH713017	MH717613	MH713189	MH713318	MH717477	-
	Col-124	Puente Genil, Córdoba, Spain; 2014	Fruit, *Olea europaea*	MH685225	MH713018	MH717614	MH713190	MH713319	MH717478	-
	Col-250	El Pedroso, Sevilla, Spain; 2011	Fruit, *Pistacia terebinthus*	MH685251	MH713019	-	-	-	-	-
	Col-332	Parga, Greece; 2012	Fruit, *Olea europaea*	MH685252	MH713020	-	-	-	-	-
	Col-338	Parga, Greece; 2012	Fruit, *Olea europaea*	MH685253	MH713021	-	-	-	-	-
	Col-347	Parga, Greece; 2012	Fruit, *Olea europaea*	MH685254	MH713022	-	-	-	-	-
	Col-350	Parga, Greece; 2012	Fruit, *Olea europaea*	MH685255	MH713023	-	-	-	-	-
	Col-378	Parga, Greece; 2012	Fruit, *Olea europaea*	MH685256	MH713024	-	-	-	-	-
	Col-384	Parga, Greece; 2012	Fruit, *Olea europaea*	MH685257	MH713025	-	-	-	-	-
	Col-388	Bari, Italy; 2012	Fruit, *Olea europea* cv. Arbosana	MH685258	MH713026	-	-	-	-	-
	Col-389	Bari, Italy; 2012	Fruit, *Olea europea* cv. Arbequina	MH685259	MH713027	-	-	-	-	-
	Col-392	Bari, Italy; 2012	Fruit, *Olea europea* cv. Cellina di Nardò	MH685261	MH713028	-	-	-	-	-
	Col-393	Bari, Italy; 2012	Fruit, *Olea europea* cv. Cellina di Nardò	MH685262	MH713029	-	-	-	-	
	Col-394	Bari, Italy; 2012	Fruit, *Olea europea* cv. Cellina di Nardò	MH685263	MH713030	-	-	-	-	-
	Col-395	Bari, Italy; 2012	Fruit, *Olea europea* cv. Ogliarola Salentina	MH685264	MH713031	-	-	-	-	-
	Col-396	Bari, Italy; 2012	Fruit, *Olea europea* cv. Ogliarola Salentina	MH685265	MH713032	-	-	-	-	-
	Col-397	Bari, Italy; 2012	Fruit, *Olea europea* cv. Cellina di Nardò	MH685266	MH713033	-	-	-	-	-
	Col-398	Bari, Italy; 2012	Fruit, *Olea europea* cv. Ogliarola Salentina	MH685267	MH713034	-	-	-	-	-
	Col-399	Bari, Italy; 2012	Fruit, *Olea europea* cv. Ogliarola Salentina	MH685268	MH713035	-	-	-	-	-
	Col-400	Bari, Italy; 2012	Fruit, *Olea europea* cv. Cellina di Nardò	MH685269	MH713036	-	-	-	-	-
	Col-454	Jerez, Cádiz, Spain; 2013	Fruit, *Olea europaea* cv. Arbequina	MH685271	MH713037	-	-	-	-	-
	Col-457	Jerez, Cádiz, Spain; 2013	Fruit, *Olea europaea* cv. Hojiblanca	MH685272	MH713038	-	-	-	-	--
	Col-462	Jerez, Cádiz, Spain; 2013	Fruit, *Olea europaea* cv. Arbequina	MH685275	MH713039	-	-	-	-	-
	Col-471	Montilla, Córdoba, Spain; 2013	Fruit, *Olea europaea* cv. Picudo	MH685277	MH713040	-	-	-	-	-
	Col-474	Montilla, Córdoba, Spain; 2013	Fruit, *Olea europaea* cv. Hojiblanca	MH685278	MH713041	-	-	-	-	-
	Col-477	Castro del Río, Córdoba, Spain; 2013	Fruit, *Olea europaea* cv. Picudo	MH685279	MH713042	-	-	-	-	-
	Col-480	Castro del Río, Córdoba, Spain; 2013	Fruit, *Olea europaea* cv. Picudo	MH685280	MH713043	MH717615	MH713191	MH713320	MH717479	-
	Col-490	Jerez, Cádiz, Spain; 2013	Fruit, *Olea europaea* cv. Hojiblanca	MH685281	MH713044	-	-	-	-	-
	Col-493	Jerez, Cádiz, Spain; 2013	Fruit, *Olea europaea* cv. Hojiblanca	MH685282	MH713045	-	-	-	-	-
	Col-499	Montilla, Córdoba, Spain; 2013	Fruit, *Olea europaea* cv. Hojiblanca	MH685283	MH713046	-	-	-	-	-
	Col-502	Fuente la Palomera, Córdoba, Spain; 2013	Fruit, *Olea europaea*	MH685284	MH713047	-	-	-	-	-
	Col-508^d,f^	Hornachuelos, Córdoba, Spain; 2014	Fruit, *Olea europaea* cv. Arbequina	KY171892	KY171900	KY171908	KY171916	KY171924	KY171932	-
	Col-511	Hornachuelos, Córdoba, Spain; 2014	Fruit, *Olea europaea* cv. Picual	MH685286	MH713048	MH717616	MH713192	MH713321	MH717480	-
	Col-512	Hornachuelos, Córdoba, Spain; 2014	Fruit, *Olea europaea* cv. Picual	MH685287	MH713049	-	-	-	-	-
	Col-514	Córdoba, Spain; 2014	Fruit, *Olea europaea* cv. Picual	MH685288	MH713050	MH717617	MH713193	MH713322	MH717481	-
	Col-515 ^f^	Córdoba, Spain; 2014	Fruit, *Olea europaea* cv. Picual	MH685289	MH713051	MH717618	MH713194	MH713323	MH717482	-
	Col-519 ^f^	Córdoba, Spain; 2014	Fruit, *Olea europaea* cv. Hojiblanca	MH685290	MH713052	MH717619	MH713195	MH713324	MH717483	-
	Col-522 ^d,f^	Lebrija, Sevilla, Spain; 2014	Fruit, *Prunus dulcis*	KY171893	KY171901	KY171909	KY171917	KY171925	KY171933	-
	Col-556	Beja, Portugal; 2014	Fruit, *Olea europaea*	MH685291	MH713053	MH717620	MH713196	MH713325	MH717484	-
	Col-558	Beja, Portugal; 2014	Fruit, *Olea europaea*	MH685292	MH713054	-	-	-	-	-
	Col-560	Beja, Portugal; 2014	Fruit, *Olea europaea*	MH685293	MH713055	-	-	-	-	-
	Col-562	Beja, Portugal; 2014	Fruit, *Olea europaea*	MH685294	MH713056	MH717621	MH713197	MH713326	MH717485	-
	Col-563	Beja, Portugal; 2014	Fruit, *Olea europaea*	MH685295	MH713057	-	-	-	-	-
	Col-564	Beja, Portugal; 2014	Fruit, *Olea europaea*	MH685296	MH713058	-	-	-	-	-
	Col-577	Montesandinha, Portugal; 2014	Fruit, *Olea europaea* cv. Arbequina	MH685301	MH713059	MH717622	MH713198	MH713327	MH717486	-
	Col-578	Capela, Portugal; 2014	Fruit, *Olea europaea* cv. Arbequina	MH685302	MH713060	MH717623	MH713199	MH713328	MH717487	-
	Col-581	Montesandinha, Portugal; 2014	Fruit, *Olea europaea* cv. Arbequina	MH685305	MH713061	MH717624	MH713200	MH713329	MH717488	-
	**CBS 133.44^T^**	Denmark	*Clarkia hybrida*	JQ948402	JQ950053	JQ949723	JQ949063	JQ949393	JQ948733	-
	**CBS 130251, OL 10, IMI 398854**	Italy	*Olea europaea*	JQ948413	JQ950064	JQ949734	JQ949074	JQ949404	JQ948744	-
	**CBS 193.32**	Greece	*Olea euroapea*	JQ948415	JQ950066	JQ949736	JQ949076	JQ949406	JQ948746	-
	**CBS 130252, IMI 398855, OL 20**	Italy	*Olea europaea*	JQ948414	JQ950065	JQ949735	JQ949075	JQ949405	JQ948745	-
	**CBS 126527, PD 93/1748**	United Kingdom	*Prunus avium*	JQ948408	JQ950059	JQ949729	JQ949069	JQ949399	JQ948739	-
	**CBS 126522, PD 88/472, BBA 70345**	The Netherlands	*Prunus cerasus*	JQ948411	JQ950062	JQ949732	JQ949072	JQ949402	JQ948742	-
	**CBS 129934, ALM-IKS-7Q**	Israel	*Prunus dulcis*	JQ948431	JQ950082	JQ949752	JQ949092.1	JQ949422	JQ948762	-
*C. guajavae*	**IMI 350839, CPC 18893^T^**	India	*Psidium guajava*	JQ948270	JQ949921	JQ949591	JQ948931	JQ949261	JQ948600	-
*C. henanense*	**LC3030, CGMCC 3.17354, LF238^T^**	China	*Camellia sinensis*	KJ955109	KJ955257	-	-	-	-	KJ954524
*C. horii*	**ICMP 10492^T^**	Japan	*Diospyros kaki*	GQ329690	JX010450	-	-	-	-	JQ807840
*C. indonesiense*	**CBS 127551, CPC 14986^T^**	Indonesia	*Eucalyptus* sp.	JQ948288	JQ949939	JQ949609	JQ948949	JQ949279	JQ948618	-
*C. jiangxiense*	**LC3463, CGMCC 3.17363, LF687^T^**	China	*Camellia. sinensis*	KJ955201	KJ955348	-	-	-	-	KJ954607
*C. johnstonii*	**CBS 128532, ICMP 12926, PRJ 1139.3^T^**	New Zealand	*Solanum lycopersicum*	JQ948444	JQ950095	JQ949435	JQ949105	JQ949105	JQ948775	-
*C. kahawae* subsp. *kahawae*	**IMI 319418, ICMP 17816**	Kenya	*Coffea arabica*	JX010231	JX010444	-	-	-	-	JQ894579
*C. karstii*	Col-79 ^d,e^	Huelva, Spain	*Citrus* sp.	MH685213	MH713151	MH713295	MH713288	MH713417	MH717492	-
	**CBS 126532**	South Africa	*Citrus* sp.	JQ005209	JQ005643	JQ005557	JQ005383	JQ005470	JQ005296	-
	**CBS 128500, ICMP 18585**	New Zealand	Fruit, *Annona cherimola*	JQ005202	JQ005636	JQ005550	JQ005376	JQ005463	JQ005289	-
	**CBS 124969, LCM 232**	Panama	Leaf, *Quercus salicifolia*	JQ005179	JQ005613	JQ005527	JQ005353	JQ005440	JQ005266	-
	**CBS 115535, STE-U 5210**	Portugal, Madeira	*Protea obtusifolia*	JQ005214	JQ005648	JQ005562	JQ005388	JQ005475	JQ005301	-
*C. kinghornii*	**CBS 198.35^T^**	United Kingdom	*Phormium* sp.	JQ948454	JQ950105	JQ949775	JQ949115	JQ949445	JQ948785	-
*C. laticiphilum*	**CBS 112989, IMI 383015, STE-U 5303^T^**	India	*Hevea basiliensis*	JQ948289	JQ949940	JQ949610	JQ948950	JQ949280	JQ948619	-
*C. limetticola*	**CBS 114.14^T^**	Florida, USA	*Citrus aurantifolia*	JQ948193	JQ949844	JQ949514	JQ948854	JQ949184	JQ948523	-
*C. lupini*	**CBS 109225; BBA 70884^T^**	Ukraine	*Lupinus albus*	JQ948155	JQ949806	JQ949476	JQ948816	JQ949146	JQ948485	-
*C. melonis*	**CBS 159.84^T^**	Brazil	*Cucumis melo*	JQ948194	JQ949845	JQ949515	JQ948855	JQ949185	JQ948524	-
*C. musae*	**ICMP 19119, CBS 116870**	USA	*Musa* sp.	JX010146	HQ596280	-	-	-	-	KC888926
*C. nymphaeae*	Col-42 ^d,e^	Tarragona, Spain, 1999	Fruit, *Olea europaea*	MH685204	MH713062	MH717625	MH713201	MH713330	MH717496	-
	Col-84 ^d,e,f^	Sevilla, Spain; 2004	Fruit, *Fragaria* × *ananassa*	MH685215	MH713063	MH717626	MH713202	MH713331	MH717497	-
	Col-86 ^d,e,f^	Sevilla, Spain; 2004	Fruit, *Fragaria* × *ananassa*	MH685216	MH713064	MH717627	MH713203	MH713332	MH717498	-
	Col-116	Montefalco, Perugia, Italy; 2014	Fruit, *Olea europaea* cv. Moraiolo	MH685222	MH713065	MH717628	MH713204	MH713333	MH717499	-
	Col-120	Navalvillar de Pela, Badajoz, Spain; 2014	Fruit, *Olea europaea* cv. Verdial de Badajoz	MH685223	MH713066	MH717629	MH713205	MH713334	MH717500	-
	Col-142	Elvas, Portugal; 2008	Fruit, *Olea euroapea*	MH685226	MH713067	-	-	-	-	-
	Col-143	Elvas, Portugal; 2008	Fruit, *Olea europaea*	MH685227	MH713068	MH717630	MH713206	MH713335	MH717501	-
	Col-150	Puebla de Guzman, Huelva, Spain; 2008	Fruit, *Olea europaea*	MH685228	MH713069	MH717631	MH713207	MH713336	MH717502	-
	Col-151	Puebla de Guzman, Huelva, Spain; 2008	Fruit, *Olea europaea*	MH685229	MH713070	-	-	-	-	-
	Col-222	Caçapava, Brasil; 2010	Fruit, *Olea europaea* cv. Arbequina	MH685246	MH713071	-	-	-	-	-
	Col-228	Uruguay; 2010	Fruit, *Olea europaea*	MH685248	MH713072	-	-	-	-	-
	Col-451	Jerez, Cádiz, Spain; 2013	Fruit, *Olea europaea* cv. Arbequina	MH685270	MH713073	MH717632	MH713208	MH713337	MH717503	-
	Col-459	Jerez, Cádiz, Spain; 2013	Fruit, *Olea europaea* cv. Hojiblanca	MH685273	MH713074	MH717633	MH713209	MH713338	MH717504	-
	Col-460	Jerez, Cádiz, Spain; 2013	Fruit, *Olea europaea* cv. Hojiblanca	MH685274	MH713075	-	-	-	-	-
	Col-466	Jerez, Cádiz, Spain; 2013	Fruit, *Olea europaea* cv. Arbequina	MH685276	MH713076	MH717634	MH713210	MH713339	MH717505	-
	Col-506 ^d,f^	Hornachuelos, Córdoba, Spain; 2014	Fruit, *Olea europaea* cv. Arbequina	KY171891	KY171899	KY171907	KY171915	KY171923	KY171931	-
	Col-510	Hornachuelos, Córdoba, Spain; 2014	Fruit, *Olea europaea* cv. Picual	MH685285	MH713077	MH717635	MH713211	MH713340	MH717506	-
	Col-572	Montesardinha,Portugal; 2014	Fruit, *Olea europaea* cv. Picual	MH685297	MH713078	MH717636	MH713212	MH713341	MH717507	-
	Col-573	Capela, Portugal; 2014	Fruit, *Olea europaea* cv. Picual	MH685298	MH713079	MH717637	MH713213	MH713342	MH717508	-
	Col-574	Montesardinha,Portugal; 2014	Fruit, *Olea europaea* cv. Arbequina	MH685299	MH713080	MH717638	MH713214	MH713343	MH717509	-
	Col-575	Montesardinha,Portugal; 2014	Fruit, *Olea europaea* cv. Picual	MH685300	MH713081	MH717639	MH713215	MH713344	MH717510	-
	Col-579	Capela, Portugal; 2014	Fruit, *Olea europaea* cv. Picual	MH685303	MH713082	MH717640	MH713216	MH713345	MH717511	-
	Col-580	Montesardinha,Portugal; 2014	Fruit, *Olea europaea* cv. Picual	MH685304	MH713083	MH717641	MH713217	MH713346	MH717512	-
	Col-615	Portugal; 2016	Fruit, *Olea europaea*	MH685306	MH713084	MH717642	MH713218	MH713347	MH717513	-
	Col-616	Portugal; 2016	Fruit, *Olea europaea*	MH685307	MH713085	MH717643	MH713219	MH713348	MH717514	-
	Col-617	Portugal; 2016	Fruit, *Olea europaea*	MH685308	MH713086	MH717644	MH713220	MH713349	MH717515	-
	Col-618	Portugal; 2016	Fruit, *Olea europaea*	MH685309	MH713087	MH717645	MH713221	MH713350	MH717516	-
	Col-619	Portugal; 2016	Fruit, *Olea europaea*	MH685310	MH713088	MH717646	MH713222	MH713351	MH717517	--
	Col-620	Portugal; 2016	Fruit, *Olea europaea*	MH685311	MH713089	MH717647	MH713223	MH713352	MH717518	-
	Col-621	Portugal; 2016	Fruit, *Olea europaea*	MH685312	MH713090	MH717648	MH713224	MH713353	MH717519	-
	Col-622	Portugal; 2016	Fruit, *Olea europaea*	MH685313	MH713091	MH717649	MH713225	MH713354	MH717520	-
	Col-623	Portugal; 2016	Fruit, *Olea europaea*	MH685314	MH713092	MH717650	MH713226	MH713355	MH717521	-
	Col-624	Portugal; 2016	Fruit, *Olea europaea*	MH685315	MH713093	MH717651	MH713227	MH713356	MH717522	-
	Col-625	Portugal; 2016	Fruit, *Olea europaea*	MH685316	MH713094	MH717652	MH713228	MH713357	MH717523	-
	Col-626	Portugal; 2016	Fruit, *Olea europaea*	MH685317	MH713095	MH717653	MH713229	MH713358	MH717524	-
	Col-627	Portugal; 2016	Fruit, *Olea europaea*	MH685318	MH713096	MH717654	MH713230	MH713359	MH717525	-
	Col-628	Portugal; 2016	Fruit, *Olea europaea*	MH685319	MH713097	MH717655	MH713231	MH713360	MH717526	-
	Col-629	Portugal; 2016	Fruit, *Olea europaea*	MH685320	MH713098	MH717656	MH713232	MH713361	MH717527	-
	Col-630	Portugal; 2016	Fruit, *Olea europaea*	MH685321	MH713099	MH717657	MH713233	MH713362	MH717528	-
	Col-631	Portugal; 2016	Fruit, *Olea europaea*	MH685322	MH713100	MH717658	MH713234	MH713363	MH717529	-
	Col-632	Portugal; 2016	Fruit, *Olea europaea*	MH685323	MH713101	MH717659	MH713235	MH713364	MH717530	-
	Col-633	Portugal; 2016	Fruit, *Olea europaea*	MH685324	MH713102	MH717660	MH713236	MH713365	MH717531	-
	Col-634	Portugal; 2016	Fruit, *Olea europaea*	MH685325	MH713103	MH717661	MH713237	MH713366	MH717532	-
	Col-635	Portugal; 2016	Fruit, *Olea europaea*	MH685326	MH713104	MH717662	MH713238	MH713367	MH717533	-
	Col-636	Portugal; 2016	Fruit, *Olea europaea*	MH685327	MH713105	MH717663	MH713239	MH713368	MH717534	--
	Col-637	Portugal; 2016	Fruit, *Olea europaea*	MH685328	MH713106	MH717664	MH713240	MH713369	MH717535	-
	Col-638	Portugal; 2016	Fruit, *Olea europaea*	MH685329	MH713107	MH717665	MH713241	MH713370	MH717536	-
	Col-639	Portugal; 2016	Fruit, *Olea europaea*	MH685330	MH713108	MH717666	MH713242	MH713371	MH717537	-
	Col-640	Portugal; 2016	Fruit, *Olea europaea*	MH685331	MH713109	MH717667	MH713243	MH713372	MH717538	-
	Col-641	Portugal; 2016	Fruit, *Olea europaea*	MH685332	MH713110	MH717668	MH713244	MH713373	MH717539	-
	Col-642	Portugal; 2016	Fruit, *Olea europaea*	MH685333	MH713111	MH717669	MH713245	MH713374	MH717540	-
	Col-643	Portugal; 2016	Fruit, *Olea europaea*	MH685334	MH713112	MH717670	MH713246	MH713375	MH717541	-
	Col-644	Portugal; 2016	Fruit, *Olea europaea*	MH685335	MH713113	MH717671	MH713247	MH713376	MH717542	-
	Col-645	Portugal; 2016	Fruit, *Olea europaea*	MH685336	MH713114	MH717672	MH713248	MH713377	MH717543	-
	Col-646	Portugal; 2016	Fruit, *Olea europaea*	MH685337	MH713115	MH717673	MH713249	MH713378	MH717544	-
	Col-647	Portugal; 2016	Fruit, *Olea europaea*	MH685338	MH713116	MH717674	MH713250	MH713379	MH717545	-
	Col-648	Portugal; 2016	Fruit, *Olea europaea*	MH685339	MH713117	MH717675	MH713251	MH713380	MH717546	-
	Col-649	Portugal; 2016	Fruit, *Olea europaea*	MH685340	MH713118	MH717676	MH713252	MH713381	MH717547	-
	Col-650	Portugal; 2016	Fruit, *Olea europaea*	MH685341	MH713119	MH717677	MH713253	MH713382	MH717548	-
	Col-651	Portugal; 2016	Fruit, *Olea europaea*	MH685342	MH713120	MH717678	MH713254	MH713383	MH717549	-
	Col-652	Portugal; 2016	Fruit, *Olea europaea*	MH685343	MH713121	MH717679	MH713255	MH713384	MH717550	-
	Col-653	Portugal; 2016	Fruit, *Olea europaea*	MH685344	MH713122	MH717680	MH713256	MH713385	MH717551	-
	Col-654	Portugal; 2016	Fruit, *Olea europaea*	MH685345	MH713123	MH717681	MH713257	MH713386	MH717552	-
	Col-655	Portugal; 2016	Fruit, *Olea europaea*	MH685346	MH713124	MH717682	MH713258	MH713387	MH717553	-
	Col-656	Portugal; 2016	Fruit, *Olea europaea*	MH685347	MH713125	MH717683	MH713259	MH713388	MH717554	-
	Col-657	Portugal; 2016	Fruit, *Olea europaea*	MH685348	MH713126	MH717684	MH713260	MH713389	MH717555	-
	Col-658	Portugal; 2016	Fruit, *Olea europaea*	MH685349	MH713127	MH717685	MH713261	MH713390	MH717556	-
	Col-659	Portugal; 2016	Fruit, *Olea europaea*	MH685350	MH713128	MH717686	MH713262	MH713391	MH717557	-
	Col-660	Portugal; 2016	Fruit, *Olea europaea*	MH685351	MH713129	MH717687	MH713263	MH713392	MH717558	-
	Col-661	Portugal; 2016	Fruit, *Olea europaea*	MH685352	MH713130	MH717688	MH713264	MH713393	MH717559	-
	Col-662	Portugal; 2016	Fruit, *Olea europaea*	MH685353	MH713131	MH717689	MH713265	MH713394	MH717560	-
	Col-663	Portugal; 2016	Fruit, *Olea europaea*	MH685354	MH713132	MH717690	MH713266	MH713395	MH717561	-
	Col-664	Portugal; 2016	Fruit, *Olea europaea*	MH685355	MH713133	MH717691	MH713267	MH713396	MH717562	-
	Col-665	Portugal; 2016	Fruit, *Olea europaea*	MH685356	MH713134	MH717692	MH713268	MH713397	MH717563	-
	Col-666	Portugal; 2016	Fruit, *Olea europaea*	MH685357	MH713135	MH717693	MH713269	MH713398	MH717564	-
	Col-667	Portugal; 2016	Fruit, *Olea europaea*	MH685358	MH713136	MH717694	MH713270	MH713399	MH717565	-
	Col-668	Portugal; 2016	Fruit, *Olea europaea*	MH685359	MH713137	MH717695	MH713271	MH713400	MH717566	-
	Col-669	Portugal; 2016	Fruit, *Olea europaea*	MH685360	MH713138	MH717696	MH713272	MH713401	MH717567	-
	Col-670	Portugal; 2016	Fruit, *Olea europaea*	MH685361	MH713139	MH717697	MH713273	MH713402	MH717568	-
	Col-671	Portugal; 2016	Fruit, *Olea europaea*	MH685362	MH713140	MH717698	MH713274	MH713403	MH717569	-
	Col-672	Portugal; 2016	Fruit, *Olea europaea*	MH685363	MH713141	MH717699	MH713275	MH713404	MH717570	-
	Col-673	Portugal; 2016	Fruit, *Olea europaea*	MH685364	MH713142	MH717700	MH713276	MH713405	MH717571	-
	Col-674	Portugal; 2016	Fruit, *Olea europaea*	MH685365	MH713143	MH717701	MH713277	MH713406	MH717572	-
	Col-675	Portugal; 2016	Fruit, *Olea europaea*	MH685366	MH713144	MH717702	MH713278	MH713407	MH717573	-
	Col-676	Portugal; 2016	Fruit, *Olea europaea*	MH685367	MH713145	MH717703	MH713279	MH713408	MH717574	-
	Col-677	Portugal; 2016	Fruit, *Olea europaea*	MH685368	MH713146	MH717704	MH713280	MH713409	MH717575	-
	Col-678	Portugal; 2016	Fruit, *Olea europaea*	MH685369	MH713147	MH717705	MH713281	MH713410	MH717576	-
	Col-679	Portugal; 2016	Fruit, *Olea europaea*	MH685370	MH713148	MH717706	MH713282	MH713411	MH717577	-
	Col-680	Portugal; 2016	Fruit, *Olea europaea*	MH685371	MH713149	MH717707	MH713283	MH713412	MH717578	-
	**CBS 515.78^T^**	The Netherlands	*Nymphaea alba*	JQ948197	JQ949848	JQ949518	JQ948858	JQ949188	JQ948527	-
	**CBS 231.49**	Portugal	*Olea europaea*	JQ948202	JQ949853	JQ949523	JQ948863	JQ949193	JQ948532	-
	**CBS 129945, PT135, RB012**	Portugal	*Olea europaea*	JQ948201	JQ949852	JQ949522	JQ948862	JQ949192	JQ948531	-
*C. orchidophilum*	**CBS 119291, MEP 1545**	Panama	*Cycnoches aureum*	JQ948154	JQ949805	JQ949475	JQ948815	JQ949145	JQ948484	-
	**CBS 632.80^T^**	USA	*Dendrobium* sp.	JQ948151	JQ949802	JQ949472	JQ948812	JQ949142	JQ948481	-
*C. paranaense*	**CBS 134729^T^**	Brazil, Parana	*Malus domestica*	KC204992	KC205060	KC205077	KC205043	KC205004	KC205026	-
*C. paxtonii*	**IMI 165753, CPC 18868^T^**	Saint Lucia	*Musa* sp.	JQ948285	JQ949936	JQ949606	JQ948946	JQ949276	JQ948615	-
*C. perseae*	Col-205; UWS-139	Australia; 2009	Fruit, *Olea europaea*	MH685242	MH713156	-	-	-	-	MH717588
	**CBS141365**	Israel	*Persea americana*	KX620308	KX620341	-	-	-	-	KX620177
*C. phormii*	**CBS 118194, AR 3546^T^**	Germany	*Phormium* sp.	JQ948446	JQ950097	JQ949767	JQ949107	JQ949437	JQ948777	-
*C. psidii*	**ICMP 19120^T^**	Italy	*Psidium* sp.	JX010219	JX010443	-	-	-	-	KC888931
*C. pyricola*	**CBS 128531, ICMP 12924, PRJ 977.1^T^**	New Zealand	*Pyrus communis*	JQ948445	JQ950096	JQ949766	JQ949106	JQ949436	JQ948776	-
*C. queenslandicum*	**ICMP 1778^T^**	Australia	*Carica papaya*	JX010276	JX010414	-	-	-	-	KC888928
*C. rhombiforme*	**CBS 129953, PT250, RB011^T^**	Portugal	*Olea europaea*	JQ948457	JQ950108	JQ949778	JQ949115	JQ949448	JQ948788	-
*C. salicis*	**CBS 607.94^T^**	The Netherlands	*Salix* sp.	JQ948460	JQ950111	JQ949781	JQ949121	JQ949451	JQ948791	-
*C. salsolae*	**ICMP 19051^T^**	Hungary	*Salsola tragus*	JX010242	JX010403	-	-	-	-	KC888925
*C. scovillei*	**CBS 126529, PD 94/921-3, BBA 70349^T^**	Indonesia	*Capsicum* sp.	JQ978267	JQ949918	JQ949588	JQ948928	JQ948928	JQ948597	-
*C. siamense*	Col-44; IMI-345047 ^d,e,f^	Spain 1999	*Fragaria vesca*	MH685205	MH713157	MH713296	MH713289	MH713418	MH717493	MH717589
	Col-160-UWS-13 ^d,e^	Australia 2009	Fruit, *Olea europaea*	MH685230	MH713158	MH713297	MH713290	MH713419	MH717494	MH717590
	Col-181; UWS-90 ^f^	Australia; 2009	Fruit, *Olea europaea*	MH685236	MH713159	-	-	-	-	MH717591
	Col-184; UWS-92 ^d,e^	Australia; 2009	Fruit, *Olea europaea*	MH685237	MH713160	-	-	-	-	MH717592
	Col-187; UWS-94 ^d,e^	Australia; 2009	Fruit, *Olea europaea*	MH685238	MH713161	-	-	-	-	MH717587
	**ICMP 18578, CBS-130417**	Thailand	*Coffea arabica*	JX010171	JX010404	-	-	-	-	JQ899289
*C. siamense* (syn. *C. jasminisambac*)	**CBS 130420, ICMP 19118**	Vietnam	*Jasminum sambac*	HM131511	JX010415	-	-	-	-	JQ807841
*C. siamense* (syn. *C. hymenocallidis*)	**CBS 125378, ICMP 18642, LC0043**	China	*Hymenocallis americana*	JX010278	JX010410	-	-	-	-	JQ899283
*C. simmondsii*	Col-169-UWS-68 ^d,e^	Australia; 2009	Fruit, *Olea europaea*	MH685232	MH713150	MH717708	MH713284	MH713413	MH717579	-
	**CBS 122122, BRIP 28519^T^**	Australia	*Carica papaya*	JQ948276	JQ949927	JQ949597	JQ948937	JQ949267	JQ948606	-
*C. sloanei*	**IMI 364297, CPC 18929^T^**	Malaysia	*Theobroma cacao*	JQ948287	JQ949938	JQ949608	JQ948948	JQ949278	JQ948617	-
*C. tamarilloi*	**CBS 129814, T.A.6^T^**	Colombia	*Solanum betaceum*	JQ948184	JQ949835	JQ949505	JQ948845	JQ949175	JQ948514	-
*C. theobromicola*	Col-200;UWS-131 ^d,e^	Australia; 2009	Fruit, *Olea europaea*	MH685241	MH713164	MH713298	MH713291	MH713420	MH717495	MH717593
	**CBS 124945^T^, ICMP 18649**	Panama	*Theobroma cacao*	JX010294	JX010447	-	-	-	-	KC790726
*C. theobromicola* (syn. *C. fragariae*)	**CBS 142.31^T^, ICMP 17927**	USA	*Fragaria ananassa*	JX010286	JX010373	-	-	-	-	JQ807844
*C. ti*	**ICMP 4832**	New Zealand	*Cordyline* sp.	JX010269	JX010442	-	-	-	-	KM360146
*C. tropicale*	**CBS 124949, ICMP 18653**	Panama	*Theobroma cacao*	JX010264	JX010407	-	-	-	-	KC790728
*C. walleri*	**CBS 125472, BMT(HL)19^T^**	Vietnam	*Coffea* sp.	JQ948275	JQ949926	JQ949596	JQ948936	JQ949266	JQ948605	-
*C. wuxiense*	**CGMCC 3.17894^T^**	China	*Camellia sinensis*	KU251591	KU252200	-	-	-	-	KU251722
*C. xanthorrhoeae*	**ICMP 17903^T^**	Australia	*Xanthorrhoea preissii*	JX010261	JX010448	-	-	-	-	KC790689

^a^ ITS: internal transcribed spacers; TUB2: beta-tubulin gene; ACT: actin gene; CHS-1: partial sequences of the chitin synthase 1; HIS3: histone H3 gene; GAPDH: 200-bp intron of the glyceraldehyde-3-phosphate dehydrogenase; ApMat: intergenic region between Apn2 and Mat1-2 genes. ^b^ Sequences from Genbank used in the phylogenetic analysis indicated in bold type; T: Isolates are ex-type or from samples that have been linked morphologically to type material of the species. ^c^ ATCC: American Type Culture Collection, Virginia, U.S.A.; CBS: Culture collection of the Centraalbureau voor Schimmelcultures, Fungal Biodiversity Centre, Utrecht, The Netherlands; IMI: Culture collection of CABI Europe UK Centre, Egham, UK; BRIP: Plant Pathology Herbarium, Department of Employment, Economic Development, and Innovation, Queensland, Australia; ICMP: International Collection of Microorganisms from Plants, Auckland, New Zealand; STE-U: Culture collection of the Department of Plant Pathology, University of Stellenbosch, South Africa; HKUCC: The University of Hong Kong Culture Collection, Hong Kong, China; PD: Plantenziektenkundige Dienst Wageningen, Nederland; UWS: University of Western Sydney; STE-U: Culture collection of the Department of Plant Pathology, University of Stellenbosch, South Africa. Sequences from GenBank used in the phylogenetic analysis indicated in bold type (Damm et al., 2012). ^d,e,f^ Representative *Colletotrichum* spp. isolates selected for morphological characterization with regards on mycelium and conidium characteristics; in vitro sensitivity tests to determine sensitivity against benomyl and ability to hydrolyse casein; and pathogenicity test to olives or to other hosts, respectively.

**Table 2:** Phenotypical characters of mycelia and conidia of representative *Colletotrichum* spp. isolates belonging to *C. acutatum*, *C. boninense*, and *C. gloeosporioides* species complexes collected from olive trees and other hosts showing anthracnose symptoms from different geographic origins.

Species Complex/Fungal Species	Isolate	Mycelium			Conidia ^d^			
		Color ^a^	Benomyl Inhibition (%) ^b^	Casein Hydrolysis ^c^	Length (µm)	Width (µm)	Length/Width	Type
***Colletotrichum acutatum* complex**								
*Colletotrichum acutatum*	Col-166/UWS-65	Pink white	33.5	+	11.1 ± 2.18	3.2 ± 0.80	3.6 ± 0.86	Ellipsoid
	Col-175/UWS-79	Pink-orange	57.4	+	8.3 ± 1.51	2.7 ± 0.58	3.1 ± 0.53	Ellipsoid
	Col-208/UWS-149	Pink gray	67.4	+	13.2 ± 2.28	4.7 ± 1.45	3.0 ± 0.71	Clavate
	Col-536	Pink-orange	*N/D*	*N/D*	10.4 ± 1.19	3.2 ± 0.62	3.3 ± 0.53	Fusiform
*Colletotrichum fioriniae*	Col-172/UWS-70	Light gray	71.1	*-*	13.4 ± 1.16	4.4 ± 0.49	3.1 ± 0.43	Fusiform
	Col-693	White	*N/D*	*N/D*	10.4 ± 0.87	3.3 ± 0.23	3.3 ± 0.32	Fusiform
	Col-694	White	*N/D*	*N/D*	10.4 ± 0.62	3.4 ± 0.23	3.2 ± 0.19	Fusiform
	Col-695	Orange gray	*N/D*	*N/D*	9.3 ± 0.55	3.2 ± 0.15	3.0 ± 0.28	Fusiform
	Col-696	Pink-orange	*N/D*	*N/D*	10.3 ± 0.45	3.8 ± 0.31	2.8 ± 0.13	Fusiform
	Col-697	White	*N/D*	*N/D*	9.9 ± 0.66	3.4 ± 0.23	3.0 ± 0.15	Fusiform
*Colletotrichum godetiae*	Col-1	Dark gray	*N/D*	*N/D*	14.8 ± 0.85	5.0 ± 0.00	3.0 ± 0.17	Clavate
	Col-9	Dark gray	55.7	++	14.8 ± 1.45	4.9 ± 0.18	3.0 ± 0.33	Clavate
	Col-30	Dark gray	63.7	++	12.5 ± 1.75	5.1 ± 0.38	2.4 ± 0.38	Clavate
	Col-50	Dark gray	59.8	++	13.9 ± 1.23	5.0 ± 0.12	2.8 ± 0.25	Clavate
	Col-51	Dark gray	67.6	++	13.5 ± 1.40	5.0 ± 0.18	2.7 ± 0.30	Clavate
	Col-52	Dark gray	*N/D*	*N/D*	13.1 ± 1.38	3.8 ± 0.19	3.5 ± 0.35	Clavate
	Col-57	Dark gray	57.2	++	13.5 ± 1.43	5.0 ± 0.18	2.7 ± 0.31	Clavate
	Col-59	Dark gray	69.0	++	13.8 ± 1.51	5.0 ± 0.18	2.8 ± 0.37	Clavate
	Col-60	Dark gray	67.0	++	14.0 ± 1.72	4.9 ± 0.38	2.9 ± 0.42	Clavate
	Col-88	Dark gray	64.5	++	12.9 ± 1.19	5.0 ± 0.04	2.6 ± 0.28	Ellipsoid
	Col-508	Dark gray	*N/D*	*N/D*	14.4 ± 1.25	3.8 ± 0.41	3.8 ± 0.47	Clavate
	Col-522	Light gray	*N/D*	*N/D*	12.8 ± 1.29	3.7 ± 0.33	3.5 ± 0.93	Fusiform
*Colletotrichum nymphaeae*	Col-42	Light gray	41.4	++	13.9 ± 1.56	3.5 ± 0.54	4.2 ± 1.06	Fusiform
	Col-84	Light gray	60.5	++	13.5 ± 1.40	3.6 ± 0.89	3.9 ± 0.35	Clavate
	Col-86	Light gray	58.7	+	14.0 ± 1.22	3.6 ± 0.43	3.9 ± 0.92	Clavate
	Col-506	Light gray	*N/D*	*N/D*	12.1 ± 1.44	3.4 ± 0.62	3.6 ± 0.57	Clavate
*Colletotrichum simmondsii*	Col-169/UWS-68	Whitish gray	64.6	+	12.4 ± 1.12	3.9 ± 0.67	3.2 ± 0.49	Fusiform
***Colletotrichum boninense* complex**								
*Colletotrichum boninense*	Col-178/UWS-82	Whitish gray	95.0	-	13.2 ± 1.02	4.8 ± 0.37	2.9 ± 0.32	Clavate
*Colletotrichum karstii*	Col-79	Pink-orange	99.4	-	12.6 ± 1.31	5.0 ± 0.02	2.6 ± 0.26	Ellipsoid
***Colletotrichum gloeosporioides* complex**								
*Colletotrichum alienum*	Col-211/UWS-152	White	94.0	++	14.1 ± 1.22	4.6 ± 0.71	3.2 ± 0.55	Ellipsoid
	Col-214/UWS-156	Pink White	95.1	-	13.9 ± 1.14	4.6 ± 0.43	3.1 ± 0.33	Ellipsoid
*Colletotrichum fructicola*	Col-82	Light gray	95.0	-	12.0 ± 1.6	3.7 ± 0.63	3.3 ± 0.56	Ellipsoid
*Colletotrichum gloeosporioides*	Col-41	Whitish gray	100	-	14.8 ± 1.45	4.4 ± 0.72	3.5 ± 0.80	Ellipsoid
	Col-69	Light gray	99.7	-	13.2 ± 1.05	5.1 ± 0.23	2.6 ± 0.24	Ellipsoid
*Colletotrichum persease*	Col-205/UWS-139	Light gray	98.2	-	14.8 ± 1.38	4.8 ± 0.86	3.2 ± 0.55	Ellipsoid
*Colletotrichum siamense*	Col-44	Green gray	96.5	-	13.9 ± 1.41	4.6 ± 0.70	3.1 ± 0.66	Clavate
	Col-160/UWS-13	Whitish gray	93.8	-	12.3 ± 1.01	4.5 ± 0.67	2.8 ± 0.50	Ellipsoid
	Col-184/UWS-92	Whitish gray	93.9	+	11.9 ± 1.38	3.5 ± 0.54	3.4 ± 0.61	Clavate
	Col-187/UWS-94	Whitish gray	96.0	++	13.3 ± 1.03	3.8 ± 0.43	3.5 ± 0.49	Ellipsoid
*Colletotrichum theobromicola*	Col-200/UWS-131	White	94.4	-	13.3 ± 2.55	4.9 ± 0.4	2.8 ± 0.48	Ellipsoid
HSD_0.05_^e^	-	-	5.67	-	1.7	0.64	0.63	

^a^ Colony color of single conidial cultures of *Colletotrichum* spp. isolates was determined on PDA by visual observations after 7 days growing at 25 ± 2 °C with a 12-h diurnal photoperiod of cool fluorescent light (350 μmol m^–2^ s^–1^). Color was determined using a color scale [42]. ^b^ Inhibition percentage (%) of mycelial growth on PDA amended with benomyl at 5 μg mL^−1^. Values represent the means of two independent experiments, each with three replicated Petri dishes per isolate. ^c^ Levels of proteolytic activity of *Colletotichum* spp. isolates: ‘-’ non-ability to hydrolyse casein; ‘+’ ability to hydrolyse casein. Presence of one or two plus symbols represents differences of halo size (‘+’: hydrolysis halo ≤ 2 mm in width; ‘++’: hydrolysis halo > 2 mm in width). Data were obtained from the means of two independent experiments, each with three replicated Petri dishes per isolate. ^d^ Conidia were obtained from colonies grown on PDA at 25 ± 2 °C with a 12-h photoperiod of fluorescent light (350 mmol m^−2^ s^−1^) for 10 days. Length and width measures and the relation between length and width (Length/Width) values represent the mean of 150 conidia ± error standard of the mean. ^e^ Critical value for comparison according to the Tukey HSD test at *p* = 0.05. *N/D* non-determined.

## Data Availability

The data that support the findings of this study are available from the corresponding author upon reasonable request.
